# Diet Quality, Nutrition Knowledge, and Social Media-Driven Supplement Use Among Polish Adolescents and Young Adults: A Cross-Sectional Study

**DOI:** 10.3390/nu18091363

**Published:** 2026-04-25

**Authors:** Klaudia Sochacka, Agata Kotowska, Sabina Lachowicz-Wiśniewska

**Affiliations:** 1 Doctoral School, The Faculty of Medicine and Health Science, University of Kalisz, W. Bogusławskiego 2, 62-800 Kalisz, Poland; szd.5.2023@uniwersytetkaliski.edu.pl; 2 Institute of Sociology, University of Rzeszów, 35-959 Rzeszów, Poland; akotowska@ur.edu.pl; 3 Department of Nutrition and Food, The Faculty of Medicine and Health Science, University of Kalisz, W. Bogusławskiego 2, 62-800 Kalisz, Poland; 4 Department of Biotechnology and Food Analysis, Wroclaw University of Economics and Business, 53-345 Wroclaw, Poland

**Keywords:** diet quality, food frequency questionnaire (FFQ), nutrition knowledge, dietary supplements, social media

## Abstract

Diet quality, nutrition knowledge, and psychosomatic literacy—defined as the understanding of the interactions between diet, gut microbiota, and mental well-being—may shape weight-related behaviours in youth. This study used a cross-sectional design to integrate these domains with digital information pathways in Central–Eastern Europe. This study assessed diet quality, nutrition, and psychosomatic knowledge, supplement use, and health-information sources among Polish adolescents and young adults, with emphasis on age-related differences and the role of social media. A cross-sectional, anonymous online survey (October 2025–January 2026) was conducted in Poland (final analytical sample: n = 478; adolescents 15–19 years vs. young adults 20–30 years). Of 591 individuals who accessed the survey, 478 were included in the final analytical sample. Diet quality was estimated from FFQ data using KomPAN-derived indices (pHDI-10, nHDI-14, DQI). Nutrition knowledge (0–25 points), psychosomatic/gut–brain indicators, supplementation, and information sources were analysed using χ^2^/Fisher tests and Mann–Whitney U tests with effect sizes. The primary outcomes measured were dietary supplement use and excess body weight (BMI ≥ 25 kg/m^2^). Multivariable logistic regression examined predictors of supplement use and BMI ≥ 25 kg/m^2^. Overall diet quality was low to moderate, with limited intake of whole grains, legumes, and fish, and common nutrition misconceptions. Social media was the most frequently indicated source of diet/supplement information and was independently associated with more frequent supplement use (OR = 2.29; 95% CI: 1.43–3.64). Adolescents reported lower whole-grain intake and more misconceptions than young adults. Predictors of BMI ≥ 25 kg/m^2^ included male sex (OR = 2.46; 95% CI: 1.46–4.15), lower education, and lower nutrition knowledge, while age showed a non-linear positive association with excess body weight. Polish adolescents and young adults show gaps between declared pro-health attitudes and actual diet quality/competencies. Social media reliance appears particularly linked to product-oriented behaviours (supplementation). Prevention should strengthen nutrition and food safety education, digital health literacy, and professional guidance on supplementation, especially in adolescents. Our findings suggest that social media is a primary driver for dietary supplementation among Polish youth, more so than objective nutrition knowledge. While diet quality is linked to weight status, the relationship is complex. These results may inform future public health interventions targeting digital health literacy to promote balanced nutrition and safe supplementation practices.

## 1. Introduction

Obesity and depression are major public health problems that increasingly co-occur across the life course. Their relationship is bidirectional: obesity has been associated with a higher risk of depression, while depressive symptoms may also increase the risk of developing obesity [1]. This coexistence is often discussed within a syndemic framework, in which biological, behavioural, and social determinants interact and mutually reinforce adverse health outcomes [2,3]. Among the mechanisms proposed to link these conditions are hypothalamic–pituitary–adrenal (HPA) axis dysregulation, chronic low-grade inflammation, altered eating behaviours, and disturbances in the gut–brain axis [4,5,6].

Growing evidence suggests that diet may play a role in both metabolic and mental health. Healthier dietary patterns have been associated with lower cardiometabolic risk and, in some studies, with improved mental well-being, although the strength and direction of these relationships remain difficult to disentangle from other lifestyle and therapeutic factors [7,8,9,10,11,12]. At the same time, increasing attention has been given to psychobiotics and microbiota-related mechanisms in obesity and depression; however, this evidence remains heterogeneous, strain-specific, and not easily translatable into everyday health behaviour [8]. Thus, although biological pathways linking obesity, depression, and the gut–brain axis are increasingly recognised, much less is known about how young people understand these issues and how such knowledge relates to their actual dietary practices.

This gap is particularly relevant in adolescents and young adults, whose dietary behaviours are increasingly shaped by digital environments. Social media and online content influence food choices, body image, supplement use, and beliefs about health, often outside professional supervision [3,13,14,15,16,17,18]. In this context, reliance on social media may be especially important for product-oriented behaviours, such as the use of dietary supplements, while its relationship with broader dietary pattern quality remains less clear. There is also limited evidence from Central and Eastern Europe, including Poland, integrating diet quality, nutrition knowledge, psychosomatic and gut–brain-related awareness, supplementation practices, and dominant sources of health information within the same analytical framework [13,14,15,16,17,18].

To address this gap, the present study examined: (i) diet quality assessed using standardised indices (pHDI-10, nHDI-14, and DQI), (ii) nutrition and psychosomatic/gut–brain-related knowledge, including awareness of the obesity–depression relationship and psychobiotics, (iii) dietary supplement use, and (iv) major sources of health information among Polish adolescents and young adults. We also compared adolescents and young adults to identify age-related differences in knowledge, behaviours, and information pathways.

**Figure 1:**
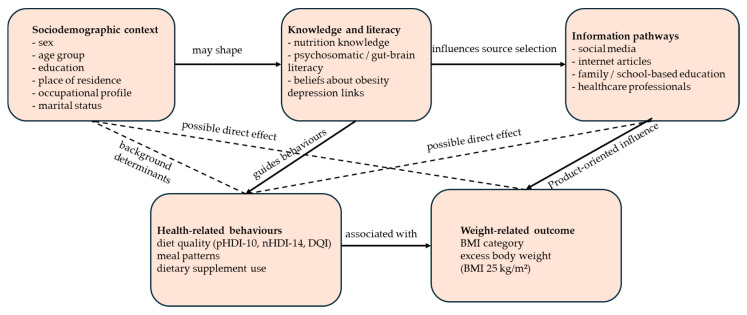
Conceptual framework of the study. The framework assumes that sociodemographic factors (sex, age group, education, place of residence, and occupational profile) shape nutrition knowledge and psychosomatic/gut–brain literacy, which in turn may influence dominant health-information pathways, particularly reliance on social media versus professional sources. These factors are expected to affect health-related behaviours, including diet quality and dietary supplement use, and ultimately weight-related outcomes, particularly excess body weight (BMI ≥ 25 kg/m^2^). The framework was used to guide the selection of variables for the multivariable models.

Therefore, the aim of this study was to assess diet quality, nutrition, and psychosomatic knowledge, dietary supplement practices, and health literacy related to obesity and depression among young adults and adolescents in Poland, with particular emphasis on their main sources of health information (social media versus professional channels). By integrating standardised diet quality indices with detailed knowledge and behaviour measures in two adjacent life-stage groups, this study may inform tailored educational and clinical strategies targeting the gut–brain axis and obesity–depression comorbidity in young populations. To strengthen the analytical framing of the study, we adopted a conceptual model linking sociodemographic characteristics, nutrition, and psychosomatic/gut–brain literacy, health-information pathways, and health-related behaviours (Figure 1). In this model, knowledge-related variables were assumed to influence both diet quality and supplementation practices, whereas reliance on social media was expected to be more strongly related to product-oriented behaviours than to overall dietary pattern quality. Sociodemographic characteristics were considered background determinants that may shape both knowledge-related competencies and behavioural outcomes, including excess body weight. Based on this framework, this study addressed the following exploratory hypotheses: (i) H1: higher nutrition knowledge and gut–brain literacy are associated with better diet quality; (ii) H2: reliance on social media as a health-information source is associated with more frequent supplement use; (iii) H3: adolescents present lower diet quality and more nutrition misconceptions than young adults; (iv) H4: sex, age, nutrition knowledge, and diet quality are associated with excess body weight.

## 2. Materials and Methods

### 2.1. Study Design and Participants

This cross-sectional study was conducted among young people in Poland using an anonymous, online diagnostic survey. Data were collected between October 2025 and January 2026. Recruitment was carried out via electronic dissemination (university mailing lists, social media, and institutional channels) to reach the target population and to reduce interviewer-related social desirability bias. All potential participants were informed about the aims and anonymous nature of the study and provided electronic informed consent prior to completing the questionnaire.

**Figure 2:**
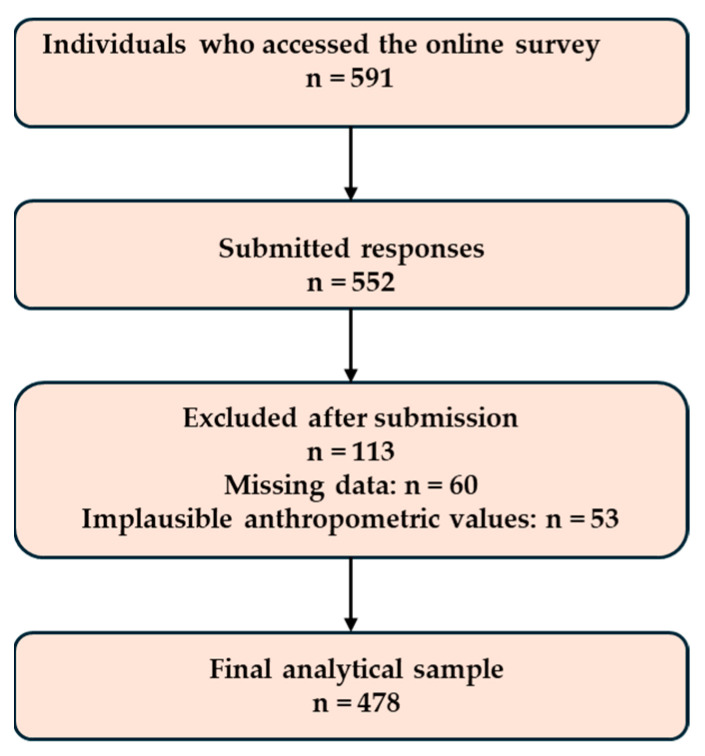
Participant flow diagram.

Inclusion criteria comprised: age (15–19 years for adolescents and 20–30 years for young adults), residence in Poland, and voluntary consent to participate (Table 1). For participants aged <18 years, electronic assent was obtained alongside electronic parental/guardian consent, in accordance with local ethical requirements. Exclusion criteria were incomplete questionnaires and implausible anthropometric data (e.g., biologically unlikely combinations of height and weight). The final analytical sample consisted of n = 478 participants. Initially, 591 individuals accessed the survey, out of which 552 submitted responses. After excluding those with missing data (n = 60) and implausible anthropometric values (n = 53), 478 cases remained for further analysis (Figure 2). To reduce duplicate entries, the survey platform restricted multiple submissions from the same device/IP where feasible, and response timestamps were screened for improbable completion times. The study protocol received approval from the Bioethics Committee of the University of Kalisz (Approval No. 20/2025) and was conducted in full accordance with the ethical standards of the Declaration of Helsinki [19]. Recruitment commenced only after ethical approval had been granted on 24 October 2025. The survey was administered via a web-based platform that allowed restriction of repeated submissions from the same device/IP where feasible; additionally, response records were screened for implausibly short completion times as a quality-control measure.

### 2.2. Survey Instrument

Data were collected using an author-designed questionnaire, structured into thematic modules and supplemented with a standardised food frequency questionnaire (FFQ). In total, the instrument comprised 60 items, allowing for a holistic assessment of sociodemographic and clinical characteristics, diet quality, health literacy, and psychosomatic awareness related to obesity and depression. The questionnaire was pre-tested on a pilot group (n = 15) to ensure linguistic clarity and ease of understanding. The internal consistency of the nutrition knowledge scale was assessed and found acceptable (Cronbach’s alpha = 0.78). Test–retest reliability was not assessed in the pilot stage. For participants aged 15–17, electronic informed consent was obtained from parents or legal guardians via a mandatory validation step in the online survey platform before the adolescent could access the questions.

The questionnaire was developed based on the KomPAN^®^ dietary habits and nutrition beliefs questionnaire prepared by the Committee of Human Nutrition Science of the Polish Academy of Sciences, with adaptation to the specific aims of this study and integration of modules on psychosomatics, supplementation, and digital health literacy. The survey was administered exclusively online (web-based questionnaire), mirroring the structure of the original KomPAN^®^ questionnaire and the FFQ but optimised for completion on computers and mobile devices [20,21,22].

The following content domains were covered:

- Sociodemographic and clinical characteristics: Age, sex, place of residence, education level, occupational profile (including sedentary and physically demanding work, shift work, and high-stress occupations), marital status, and self-reported chronic conditions, with particular attention to endocrine and cardiometabolic disorders. Body mass index (BMI, kg/m^2^) was calculated from self-reported body weight and height, and further categorized into underweight, normal weight, overweight, and obesity classes according to WHO criteria.
- Dietary habits and theoretical nutrition knowledge: regularity and structure of meals (including breakfast consumption), perceived energy balance, and identification of common nutrition myths (e.g., the role of saturated fats, cholesterol, and perceived deficiencies in elimination diets).
- Food frequency questionnaire (FFQ): Qualitative assessment of the frequency of consumption of major food groups, including whole-grain products, refined cereals, dairy products, white and red meat, processed meat, fish, legumes, vegetables, fruit, confectionery, sugar-sweetened beverages, fast food, and ultra-processed foods. FFQ responses were converted into daily consumption frequencies, summed within pro-healthy (10 food groups) and non-healthy (14 food groups) categories to obtain pHDI-10 and nHDI-14, and then expressed on a 0–100 scale according to the KomPAN manual. Index values were interpreted as low (0–33), moderate (34–66) or high (67–100).
- Food safety and hygiene: knowledge of microbiological and chemical hazards in food (e.g., mycotoxins in mouldy bread), understanding of product labelling and expiry dates, and self-reported practices related to reading and interpreting food labels.
- Supplementation and phytotherapy: use of dietary supplements and herbal preparations, motives for supplementation, perceived indications and contraindications, and sources of recommendations, with particular emphasis on products marketed for weight management (e.g., berberine, chromium, white mulberry, green tea extracts) and sport-oriented preparations (protein supplements, creatine).
- Psychosomatics of obesity and the gut–brain axis: awareness of the bidirectional relationship between depression and body weight, knowledge of probiotic therapy and psychobiotics, and the ability to recognise affective and somatic manifestations of mood disorders (e.g., gastrointestinal complaints, sleep disturbances, fatigue).
- Sources of health and nutrition information: main channels used to obtain information on diet, obesity, and mental health (social media platforms, influencers, internet articles, traditional media, school-based education, healthcare professionals, and public health campaigns).

To ensure high data quality, the questionnaire included filtering and control questions that enabled detection of inconsistent or random responding. Self-reported height and weight were cross-checked for plausibility before BMI calculation and further analyses. Items assessing specialised knowledge (e.g., recognition of psychobiotic strains or somatic symptoms of depression) were formulated as open- or multiple-choice questions, enabling a more accurate evaluation of actual health competencies rather than mere familiarity with terminology.

### 2.3. Statistical Analysis

All statistical analyses were performed using Statistica 13.3 (StatSoft, Tulsa, OK, USA) and Microsoft Excel 2019 (Microsoft Corp., Redmond, WA, USA). The level of statistical significance was set at *p* < 0.05 for all tests, and two-tailed *p*-values are reported.

Continuous variables were first inspected for distributional properties using measures of central tendency, dispersion, and skewness. As most variables deviated from a normal distribution and exhibited skewness, data are presented as the median and interquartile range (Q1–Q3), together with minimum and maximum values and skewness coefficients for the total sample and stratified by sex. Categorical variables are reported as counts (n) and percentages (%).

Between-group comparisons of continuous variables (e.g., age, BMI, pHDI, nHDI, DQI, nutrition knowledge score) by sex were conducted using the Mann–Whitney U test, given the non-normal distribution of the data. For these analyses, effect sizes were quantified using the rank-biserial correlation coefficient r with 95% confidence intervals and Cliff’s delta (δ) to provide an estimate of the magnitude of differences beyond *p*-values.

Associations between categorical variables (e.g., BMI categories, education level, place of residence, occupational profile, marital status, diet quality profiles, knowledge levels) and grouping variables were examined using chi-square ($\chi^{2}$) tests of independence. When expected cell counts were small, Fisher’s exact test was applied as appropriate. The strength of associations for contingency tables was expressed using Cramér’s V coefficient, with corresponding interpretations of effect size.

Diet quality profiles were derived from the pro-healthy diet index (pHDI10) and non-healthy diet index (nHDI14), calculated from FFQ data according to established procedures. Based on standard cut-offs for low, moderate, and high values of pHDI and nHDI in the sample, participants were classified into combined DQI profiles reflecting different patterns of diet quality (e.g., low pHDI/low nHDI, moderate pHDI/low nHDI). The distribution of sociodemographic characteristics, BMI categories, and levels of nutrition knowledge across these DQI profiles was compared using chi-squared tests for categorical variables and Kruskal–Wallis tests for continuous variables. For Kruskal–Wallis analyses, epsilon-squared (ε^2^) effect sizes were reported to quantify the proportion of variance in the outcome attributable to group differences. Effect size magnitude was interpreted using conventional thresholds for non-parametric analyses [23]. An ordinal logistic sensitivity analysis using three outcome categories (no use/occasional use/regular use) was additionally performed to evaluate the robustness of the binary supplementation model.

To identify independent predictors of the two primary outcomes, two multivariable logistic regression models were constructed: one for dietary supplement use (yes/sometimes vs. no) and one for excess body weight (normal weight vs. overweight/obesity; BMI ≥ 25 kg/m^2^). Candidate predictors for the multivariable models were selected a priori according to the study’s conceptual framework, which assumed that sociodemographic characteristics may shape knowledge-related competencies and health-information pathways, and that these factors, in turn, may influence diet quality, supplementation practices, and excess body weight. Accordingly, variables representing background characteristics (sex, age, place of residence, education level, occupational profile, and marital status), knowledge-related competencies (nutrition knowledge score), behavioural indicators (pHDI-10 and nHDI-14 in the BMI model; DQI in the supplementation model), and information pathway variables (social media as a source of information in the supplementation model) were entered into the regression analyses. In the model predicting supplement use, social media reliance was treated as a theoretically relevant exposure variable because the conceptual framework assumed that digital information pathways may be more strongly linked to product-oriented behaviours than to broader dietary pattern quality. For the primary logistic regression analysis, regular supplement use and occasional supplement use were combined into a single category to distinguish participants with any active supplementation behaviour from non-users. This binary operationalisation was chosen to reflect the presence versus absence of supplementation behaviour in the main model, while an ordinal sensitivity analysis was additionally performed to assess whether the results were robust to a more granular outcome definition.

A nutrition knowledge score was computed by awarding 1 point for each correct response (0 for incorrect or “don’t know”), yielding a total range of 0–25; higher scores indicated greater knowledge. Odds ratios (ORs) with 95% confidence intervals were estimated for each predictor, and statistical significance was evaluated using Wald tests. Multicollinearity was assessed using variance inflation factors (VIFs). Linearity of the logit for continuous predictors was confirmed using the Box–Tidwell approach. Model fit was assessed using the Hosmer–Lemeshow test, and model discrimination was evaluated using the area under the receiver operating characteristic curve (AUC), with results reported in the Results Section. Because assessment of the linearity of the logit indicated a non-linear relationship for age in the BMI ≥ 25 kg/m^2^ model, age was modelled in the final regression using centred linear and quadratic terms. This specification improved model fit and was retained in the final analysis.

As a post hoc justification of sample adequacy, the model for dietary supplement use included 378 events and 100 non-events for six predictors, corresponding to 63.0 events per predictor. The model for excess body weight (BMI ≥ 25 kg/m^2^) included 115 events for nine predictors, corresponding to 12.8 events per predictor. Both values exceeded commonly recommended thresholds for logistic regression modelling. In addition, given N = 478, the observed outcome prevalences, and α = 0.05, the study had approximately 80% power to detect odds ratios of about 1.9 or greater for binary predictors with prevalences similar to the main exposures analysed.

**Exploratory cluster analysis of dietary habits:** Additionally, an exploratory cluster analysis was performed to identify data-driven dietary-behaviour profiles within the study population. The analysis was based on standardized dietary indicators derived from the FFQ and selected meal-pattern variables, including pHDI-10, nHDI-14, eating fewer than three meals per day, eating before sleep, snacking between meals, eating in a hurry, purchasing ready-made meals, and fast-food consumption. All variables were z-standardized before clustering. A k-means clustering procedure was applied, and a three-cluster solution was retained based on interpretability and balanced cluster sizes. The resulting clusters were then compared with respect to sociodemographic characteristics, BMI category, nutrition knowledge, gut–brain-related literacy, supplementation practices, and selected health-information variables using chi-square tests for categorical variables and Kruskal–Wallis tests for continuous or ordinal variables.

## 3. Results

### 3.1. Characteristics of the Study Population

The final sample included 478 respondents, predominantly women (65.7%), with a median age of 20.0 years (Q1–Q3: 18.0–25.0; range 17.0–30.0 years) (Table 1 and Table 2). The median BMI in the total group was 22.05 kg/m^2^ (Q1–Q3: 19.95–24.84), and 62.8% of participants were classified as having normal weight, 16.5% as overweight, and 7.6% as obese (classes I–III combined) (Table 1, Table 2 and Table 3). Women had a significantly lower median BMI than men (21.22 vs. 23.55 kg/m^2^; *p* < 0.0001), while men reported higher nHDI-14 values and women showed higher DQI values; pHDI-10 did not differ significantly by sex (Table 4).

Perceived body weight differed significantly across BMI categories (Table S1; χ^2^ *p* < 0.001; Cramér’s V = 0.513). Among underweight participants (n = 63), 50.8% perceived their body weight as too low, whereas 34.9% perceived it as normal. In the normal-weight group, 70.0% perceived their body weight as normal, while misperception was also present in higher-BMI categories: 30.4% of overweight participants perceived their weight as normal, and only 55.6% of participants with obesity perceived themselves as obese.

The underweight subgroup (BMI < 18.5 kg/m^2^; 13.2% of the sample) was predominantly female (16.6% of all women vs. 6.7% of all men). In this subgroup, 64.5% reported regular or occasional dietary supplement use. Their nutrition knowledge scores were comparable to those of the normal-weight group (median 11 points), but they more frequently indicated social media as a source of health information (72%). Self-reported chronic conditions were reported by 16.1% of participants (77/478). The most frequently indicated categories were thyroid disorders (6.3%), respiratory/allergic disorders (3.1%), hypertension (2.1%), and diabetes or insulin resistance (1.7%) (Table S11).

The skewness coefficients shown in Table 2 supported the use of non-parametric analyses.

### 3.2. Diet Quality and Nutrition Knowledge

Median values of the pro-healthy diet index (pHDI-10) and non-healthy diet index (nHDI-14) in the total sample were 20.60 (Q1–Q3: 12.68–31.30) and 15.86 (Q1–Q3: 10.57–22.29) points, respectively, indicating generally low intensity of both dietary components with their concurrent presence in eating patterns (Table 2). Median DQI was 5.06 points (Q1–Q3: −4.72–16.49), ranging from −83.34 to 50.63, which reflects substantial inter-individual variability in overall diet quality (Table 2). Women and men did not differ significantly in pHDI-10; however, men showed higher nHDI-14 values (*p* < 0.001; r = 0.268; Cliff’s delta (δ) = −0.326), corresponding to a less favourable dietary profile (Table 4).

The median nutrition knowledge score was 11.0 points (Q1–Q3: 9.0–14.0) out of 25, corresponding to 44% (Q1–Q3: 36–56%) of correct responses (Table 2). Women achieved slightly higher knowledge scores than men, although the effect size was small (*p* = 0.0249; r = 0.102; δ = 0.124) (Table 4). According to KomPAN thresholds, 20.1% of respondents had insufficient knowledge (0–8 points), 68.2% sufficient knowledge (9–16 points), and 11.7% good knowledge (17–25 points) (Table 5). Diet quality profiles were associated with place of residence (*p* = 0.0031; Cramér’s V = 0.155) and nutrition knowledge level (*p* = 0.0182; Cramér’s V = 0.112), with a stronger association observed for the KomPAN proposal I knowledge profile (*p* = 0.0001; Cramér’s V = 0.170) (Table 5). BMI also differed across DQI profiles (*p* = 0.0042; ε^2^ = 0.019), although the effect size was small (Table 5).

### 3.3. Diet Quality Profiles and Their Determinants

Based on combined pHDI-10 and nHDI-14 categories, five diet quality profiles were identified (Table 6). The predominant pattern was characterised by concurrently low pHDI-10 and low nHDI-14 (Profile 1; 70.7%), indicating low overall intensity of both recommended- and non-recommended-food consumption. Profiles reflecting low pHDI-10 with moderate or high nHDI-14 were uncommon (Profile 2: 6.7%; Profile 3: 0.4%), whereas 20.1% of participants showed a more favourable pattern with moderate pHDI-10 and low nHDI-14 (Profile 4); only 2.1% presented moderate pHDI-10 together with moderate nHDI-14 (Profile 5) (Table 6). When aggregated into three overall DQI categories, Profile 2 and Profile 3 jointly formed the low-diet-quality group (L_DQI; 7.1%), Profile 1 corresponded to moderate diet quality (M_DQI; 70.7%), and Profile 4 plus Profile 5 constituted the high-diet-quality group (U_DQI; 22.2%) (Table 5).

Higher overall diet quality (U_DQI) was associated with place of residence (urban: 56.6% in U_DQI vs. 23.5% in L_DQI; *p* = 0.0031; Cramér’s V = 0.155) and nutrition knowledge (KomPAN knowledge level: *p* = 0.0182; V = 0.112; knowledge profile proposal I: *p* = 0.0001; V = 0.170) (Table 5). Participants in U_DQI also achieved higher knowledge scores (median 13.0 vs. 10.0–11.0 points; *p* = 0.0001; ε^2^ = 0.033) and exhibited slightly higher BMI compared with lower-DQI categories (median 23.55 vs. 21.33–21.83 kg/m^2^; *p* = 0.0042; ε^2^ = 0.019), although the effect sizes were small (Table 5). A trend towards higher diet quality with tertiary education was observed but did not reach conventional statistical significance (*p* > 0.05) (Table 5).

Item-level analyses further indicated fragmented nutritional knowledge: while 87.6% correctly recognised the role of vegetables in a healthy diet, 51.1% misjudged the recommended intake of cereal products, and 32.0% believed that protein should be the main energy source. Misconceptions regarding lipids and food safety were also common, including beliefs that vegetable oils contain cholesterol (28.3%) and that vegetarian diets inevitably lead to anemia (69.0%); only 26.9% correctly identified mouldy bread as a source of mycotoxins rather than Salmonella contamination.

Cluster analysis of dietary habits: An exploratory cluster analysis identified three distinct dietary-behaviour profiles in the study population (Table 7). Cluster 1, termed the higher-pro-healthy/relatively regular pattern (n = 150; 31.4%), was characterized by the highest pHDI-10 values and relatively regular meal habits, with lower frequency of eating fewer than three meals per day and moderate levels of unhealthily food consumption. Cluster 2, termed the lower-unhealthy/lower-intensity pattern (n = 140; 29.3%), showed low-to-moderate pHDI-10 together with the lowest nHDI-14 values, indicating generally lower intensity of both healthy and unhealthy food consumption. Cluster 3, termed the unhealthy–irregular pattern (n = 188; 39.3%), was characterized by the highest nHDI-14 values and the greatest frequency of irregular eating behaviours, including snacking, eating before sleep, eating in a hurry, purchasing ready-made meals, and fast-food consumption.

The clusters differed significantly in nutrition knowledge (*p* < 0.001), gut–brain literacy (*p* < 0.001), BMI category (*p* < 0.001), supplementation practices (*p* < 0.001), sex distribution (*p* = 0.005), and age-group distribution (*p* = 0.008). Participants in the unhealthy–irregular cluster had the lowest median nutrition knowledge score and the lowest gut–brain literacy. Underweight was least frequent in the higher-pro-healthy/relatively regular cluster (5.3%) and more common in the lower-unhealthy/lower-intensity and unhealthy–irregular clusters (16.4% and 17.0%, respectively). Any supplement use was highest in the lower-unhealthy/lower-intensity cluster (88.6%) and lowest in the unhealthy–irregular cluster (71.3%). In contrast, the proportion of participants reporting social media as a source of diet/supplement information did not differ materially across clusters (all approximately 56–57%).

### 3.4. Eating Patterns, Supplement Use, and Sources of Information

Meal-pattern analysis showed that 69.0% of participants consumed a warm meal daily or almost daily, and 25.5% did so several times per week. Irregular eating behaviours were also common: 33.1% reported consuming fewer than three meals per day several times per month and 19.0% several times per week, while approximately half of the sample reported regular breakfast skipping. Eating in a hurry and consuming meals directly before sleep were also frequently reported.

Dietary intake patterns were characterised by a predominance of refined products over whole-grain foods. Wholemeal bread and whole-grain cereals were consumed relatively infrequently, whereas white bread and refined grain products were consumed more often. Fish and legumes were also consumed infrequently, most commonly only 1–3 times per month. In contrast, white meat, eggs, milk, fermented dairy products, sweets, and processed foods were consumed more regularly. Convenience foods, snacking between meals, and fast-food intake were also common.

Supplement use was frequent (Table S2): 66.9% of participants reported regular use and 12.1% occasional use, with significant differences between adolescents and young adults (χ^2^ *p* = 0.0047; Cramér’s V = 0.150). Young adults more often reported regular use, whereas adolescents more often reported occasional use. Among supplement users, self-directed supplementation predominated (69.8%), while physician-only and dietitian-only recommendations were less common (18.0% and 6.3%, respectively).

Social media was the most frequently indicated source of information on diet and supplementation (56.9%), followed by family (46.9%) and internet articles (43.1%) (Table S2). Adolescents more often indicated family, whereas young adults more often indicated internet articles. Selecting social media as an information source was associated with more frequent supplement use (χ^2^ *p* = 0.0049; Cramér’s V = 0.129) (Table 8). Social media selection was not associated with pHDI-10, nHDI-14, or DQI, while a small difference was observed for nutrition knowledge score (*p* = 0.0335). In multivariable logistic regression, social media reliance remained independently associated with supplement use (OR = 2.29, 95% CI: 1.43–3.64; *p* = 0.0005), and a higher nutrition knowledge score was also an independent predictor (OR = 1.09, 95% CI: 1.02–1.16; *p* = 0.0111), whereas sex, age, education, and DQI were not significant (Table 8). Model diagnostics indicated acceptable fit for the supplementation model. The Hosmer–Lemeshow test was non-significant (χ^2^ = 8.92, *p* = 0.349), Nagelkerke’s R^2^ was 0.076, and discrimination was modest but acceptable (AUC = 0.664, 95% CI: 0.599–0.722). Multicollinearity was low (all VIF values 1.05–1.45). Box–Tidwell testing did not indicate meaningful deviations from linearity of the logit for age, nutrition knowledge score, or DQI (all *p* > 0.05). In an ordinal logistic sensitivity analysis using three outcome categories (no use/occasional use/regular use), the main conclusions remained unchanged. Social media as a source of information was positively associated with higher supplementation frequency (OR = 2.05, 95% CI: 1.37–3.06; *p* < 0.001), and a higher nutrition knowledge score was also positively associated with supplementation frequency (OR = 1.12, 95% CI: 1.06–1.19; *p* < 0.001), whereas sex, age, education, and DQI were not significant.

### 3.5. Comparative Analysis of Adolescents and Young Adults

Comparative analysis of FFQ data indicated age-related differences in dietary choices. Adolescents reported lower intake of whole-grain products: 20.2% declared never consuming wholemeal bread, and 36.2% consumed whole-grain groats or pasta only 1–3 times per month; in young adults, these products were more often consumed several times per week (Table S6). Legume intake was also low among adolescents, with 19.1% reporting no consumption and 47.9% reporting intake only 1–3 times per month. Fish consumption was generally low in both groups; however, adolescents most frequently reported eating fish 1–3 times per month (57.4%), and 13.8% reported never consuming fish (Table S6).

Adolescents also reported frequent consumption of white meat (59.6% several times per week) and daily milk intake (26.6%), while fermented dairy products were consumed less frequently than in young adults (11.7% once daily). Fruit consumption was reported daily by 34.0% of adolescents (Table S6).

Nutrition knowledge among adolescents showed substantial misconceptions. Nearly 60% (59.6%) believed that eating mouldy bread could result in Salmonella infection, and 54.3% indicated that protein should be the primary energy source in a proper diet. Misconceptions regarding dietary fats were also common: 45.7% believed that oils and olive oils contain large amounts of cholesterol, and 47.9% were unable to clearly accept or reject this statement. Regarding fish, only 21.3% correctly rejected the statement that frequent consumption of fatty sea fish accelerates atherosclerosis, while 58.5% reported no opinion (Table S7).

Additionally, 61.7% of adolescents considered anaemia risk an inevitable consequence of vegetarian diets; only 10.6% correctly identified this statement as false, while 27.7% were unsure (Table S7).

### 3.6. Psychosomatic Awareness, Supplementation, and Gut–Brain-Related Knowledge

Indicators of psychosomatic and gut–brain-related knowledge are summarised in Table S5. Most respondents endorsed the obesity–depression link (81.6%) and reported that diet can influence gut microbiota composition (82.0%). Self-declared knowledge of probiotics/psychobiotics was high (78.7%), and 72.4% believed that these interventions may improve mood. However, operational knowledge was limited: only 13.8% reported knowing typical psychobiotic strains, whereas 67.4% reported no knowledge in this area; uncertainty was also evident for food-based sources, as 30.1% were unsure whether bio-yoghurts contain beneficial gut bacteria (Table S5).

Self-rated mental well-being was most frequently reported as good (58.2%), while 19.7% reported an unsatisfactory level (Table S8). Most respondents endorsed the role of dietary habits in disease prevention/development (89.5%), with slightly higher endorsement among young adults than adolescents (92.6% vs. 86.4%; *p* = 0.0417; Cramér’s V = 0.093). Almost all participants considered mental health important for a healthy lifestyle (96.7%). A majority agreed that moderate physical activity may reduce depressive symptoms (75.7%) and that food affects mood/emotions (94.6%) (Table S8).

Depression literacy indicators are presented in Table S9. Only half of respondents declared that they could recognise depression in themselves or a close person (50.6%), while 40.2% reported uncertainty. Depression was widely perceived as a public health problem in Poland (76.6%), with no significant differences by age group (*p* = 0.5178). In open-ended responses, affective descriptors predominated (apathy/anhedonia: 78.2%; sadness/low mood: 74.5%), whereas somatic/gastrointestinal manifestations were rarely mentioned (2.9%) (symptom coding in Supplementary Table S9). Sleep problems (35.1%) and fatigue (26.4%) were the most frequently reported non-affective symptom categories.

Supplement-related behaviours and information pathways are presented in Table 8 and are described in detail in Section 3.4; here, we focus on psychosomatic, gut–brain-related, and depression literacy indicators.

**Figure 3:**
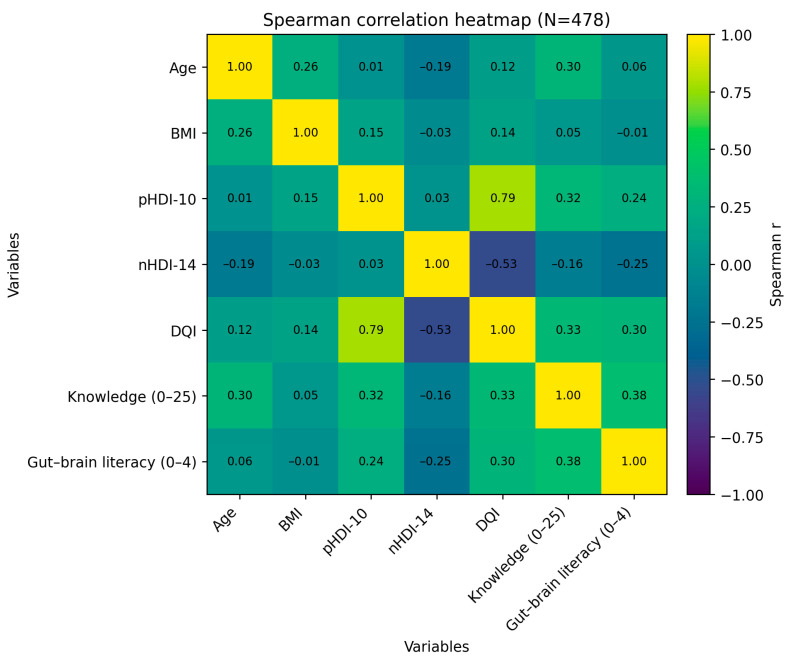
Spearman correlation heatmap (n = 478) for age, BMI, diet quality indices (pHDI-10, nHDI-14, DQI), nutrition knowledge (0–25), and gut–brain literacy (0–4). Values in cells represent Spearman’s r. Given the sample size, correlations with |r| ≥ 0.12 were generally significant at *p* < 0.01.

Correlation analysis is shown in Figure 3. Overall diet quality (DQI) was strongly positively associated with pHDI-10 (r = 0.79) and moderately negatively associated with nHDI-14 (r = −0.53), consistent with the construction of DQI. Nutrition knowledge (0–25) correlated moderately with DQI (r = 0.33) and pHDI-10 (r = 0.32), and weakly negatively with nHDI-14 (r = −0.16). Gut–brain literacy (0–4) correlated moderately with nutrition knowledge (r = 0.38) and DQI (r = 0.30), and negatively with nHDI-14 (r = −0.25). Age showed a modest positive correlation with BMI (r = 0.26) and nutrition knowledge (r = 0.30), while BMI was only weakly related to diet quality indices (|r| ≤ 0.15); given the sample size (n = 478), correlations with |r| ≥ 0.12 were generally statistically significant at *p* < 0.01.

### 3.7. Predictors of Excess Body Weight

To identify factors independently associated with excess body weight, a multivariable logistic regression model was fitted with BMI dichotomised as normal weight vs. BMI ≥ 25 kg/m^2^ (Table 9). Male sex remained positively associated with excess body weight (OR = 2.46, 95% CI: 1.46–4.15; *p* = 0.0008), whereas higher education was inversely associated with BMI ≥ 25 kg/m^2^ (OR = 0.21, 95% CI: 0.09–0.45; *p* < 0.001). Greater nutrition knowledge was also inversely associated with excess body weight (OR = 0.92, 95% CI: 0.86–0.99; *p* = 0.021), while pHDI remained positively associated with BMI ≥ 25 kg/m^2^ (OR = 1.04, 95% CI: 1.02–1.06; *p* < 0.001). Place of residence, sedentary work, marital status, and nHDI were not significant in the final model. Age showed a non-linear positive association with BMI ≥ 25 kg/m^2^ and was therefore modelled using centred linear and quadratic terms.

Model diagnostics indicated acceptable fit for the final BMI model. The Hosmer–Lemeshow test was non-significant (χ^2^ = 11.84, *p* = 0.159), Nagelkerke’s R^2^ was 0.268, and discrimination was acceptable (AUC = 0.777). Multicollinearity was low for the main dietary predictors (pHDI VIF = 1.13; nHDI VIF = 1.14), indicating that problematic collinearity between these indices was unlikely.

## 4. Discussion

This study revealed a pronounced discrepancy between declarative pro-health attitudes and the actual diet quality and health competencies of Polish adolescents and young adults. Although most respondents recognised the importance of diet for health and reported frequent consumption of warm meals, their overall diet quality was only low to moderate, with predominant intake of refined cereals, limited consumption of fish and legumes, and common snacking on sweets and salty products. The median nutrition knowledge score corresponded to 44% correct answers, and the majority of participants achieved only a “sufficient”, not “good”, level of knowledge. Similar gaps between awareness and behaviour have been described in Polish adolescents and young adults, who often fail to meet national food-based dietary guidelines, particularly with respect to whole-grain products, legumes, and fish [3,11,24,25].

A central finding of this work is the dominance of social media and online content as primary sources of nutrition and health information. This pattern is consistent with recent observational and systematic studies showing that adolescents and young adults increasingly rely on digital platforms for dietary advice, which is frequently driven by commercial interests rather than evidence-based recommendations. In our cohort, such reliance co-occurred with persistent nutrition myths, including beliefs that protein should be the main energy source, that vegetable oils contain cholesterol, and that vegetarian diets inevitably lead to anaemia. Comparable misconceptions about lipids, plant-based diets, and “high-protein” regimens have been documented in European youth and young adults, where social media often amplifies simplified or sensationalised narratives. Together with the modest overall knowledge scores, these findings underscore the importance of digital health literacy as a determinant of both diet quality and weight-related behaviours in this age group [3,13,14,16,26,27,28]. Importantly, our results suggest that the behavioural correlate of social media reliance was most evident for supplementation practices (higher odds of supplement use), whereas no clear association was observed with overall diet quality indices (pHDI-10/nHDI-14/DQI). This pattern indicates that online content may shape product-oriented decisions more strongly than broad dietary pattern change.

The dietary pattern identified in this study—high consumption of white bread and processed meats, and low consumption of whole grains, legumes, and fish—mirrors the Westernised diet associated in numerous studies with increased risk of obesity, cardiometabolic disease, and depressive symptoms. Polish dietary guidelines explicitly recommend regular intake of whole-grain cereals, legumes, and fatty sea fish as key sources of fiber, plant protein, iron, B vitamins, and long-chain omega-3 fatty acids, yet our results show that this food groups remain markedly underrepresented in everyday diets. The very low frequency of fish consumption and limited awareness of the cardioprotective role of marine n-3 fatty acids, especially among adolescents, are particularly concerning in light of evidence linking higher intake of these nutrients with lower risk of both cardiovascular disease and depressive symptoms. This pattern is consistent with previous Polish data showing low adherence to recommendations for fish intake in this age group [29,30]. At the same time, the rare use of lard and relatively frequent consumption of fermented dairy products suggest that some potentially beneficial elements of traditional dietary patterns are maintained, providing culturally meaningful entry points for interventions [3,12,25,31,32,33]. Additionally, the observed misperception of body weight status (including a substantial proportion of participants with overweight perceiving their weight as normal and only about half of those with obesity perceiving themselves as obese) may hinder timely engagement in evidence-based weight management and increase vulnerability to simplified online narratives and supplement-focused strategies (Table S1). Our data also highlight important deficiencies in food safety knowledge. Only about one quarter of respondents correctly recognised that mould on bread signifies mycotoxin production rather than Salmonella contamination, despite clear public health guidance that mouldy bread should be discarded because mycotoxins can diffuse beyond visible growth. Experimental studies have confirmed that bread may contain various mycotoxins and that quantifying exposure is challenging due to heterogeneous contamination. The low level of awareness observed in our sample, combined with other microbiological misconceptions, suggests insufficient emphasis on food hygiene and basic food microbiology within current health education, and underscores the need to integrate these topics into broader health literacy initiatives [34,35].

With respect to supplementation, our findings align with emerging evidence of widespread, largely unsupervised use of dietary supplements among young adults. In our cohort, adults most often used phytotherapeutic and nutraceutical agents aimed at carbohydrate metabolism, such as berberine, white mulberry, and chromium, whereas adolescents more frequently reached for sport-oriented products such as protein supplements and creatine, which may reflect a preference for performance- or body-composition-oriented approaches to weight-related goals rather than structured dietary change alone. Existing studies suggest that berberine and chromium can modestly improve glycaemic control and lipid profiles, but their long-term safety, potential interactions, and real-world effectiveness, particularly in polypharmacy contexts, remain incompletely characterised. The very low rate of professional consultation about supplement use in our sample is consistent with European data and raises concerns, especially in the presence of self-reported endocrine or metabolic comorbidities. These observations support calls for closer collaboration between dietitians, physicians, and pharmacists in counselling young people about weight management and mental health [9,10,12]. In line with this, our analyses showed that social media reliance was independently associated with supplement use, reinforcing the need to frame supplement counselling within digital health literacy efforts (Table 8).

The present study extends the existing literature by introducing a psychosomatic and gut–brain axis perspective into the analysis of youth diet and health literacy. Most respondents acknowledged a bidirectional relationship between obesity and depression and expressed belief in the potential of psychobiotics, yet their operational knowledge remained limited. Recent systematic reviews suggest that selected probiotic strains may modestly reduce depressive symptoms, but the evidence is heterogeneous and strongly strain-dependent. The coexistence in our cohort of high declarative enthusiasm for psychobiotics with poor knowledge of specific strains underscores the need for carefully framed communication about realistic expectations for probiotic interventions in mood disorders [33,34,36]. Participants also showed difficulty recognising somatic manifestations of depression, focusing primarily on affective symptoms while rarely mentioning gastrointestinal complaints, sleep disturbances, or fatigue. This pattern may delay recognition of mood disorders and reduce the effectiveness of integrated dietary and psychological interventions. Our results therefore support the value of integrated care models in which dietitians, psychologists, and physicians jointly address metabolic and mental health dimensions of obesity [3,37,38,39,40,41]. The comparative analysis between adolescents and young adults adds a life-course perspective that has been less visible in previous work. Earlier studies in Polish adolescents have documented low adherence to recommendations for whole grains, milk, and fish, with particularly poor intake of foods supplying calcium, fibre, and omega-3 fatty acids. Our findings extend this evidence by showing that adolescents not only consume fewer whole-grain products, legumes, and fish than young adults, but also exhibit a higher prevalence of specific nutrition myths, including exaggerated fear of anaemia on vegetarian diets and confusion about the lipid profile of vegetable oils. These deficits cluster precisely in domains central to the prevention of both obesity and depression, suggesting that early, targeted education on plant-based sources of iron and B vitamins, as well as on marine fats and fibre, is critical. Although young adults in our sample displayed slightly better diet quality and knowledge, their overall competencies still appear insufficient to ensure effective self-management of weight and mental health [3,32].

The logistic regression model further clarified the profile of individuals at higher risk of excess body weight in this young population. Male sex and lower education were associated with greater odds of overweight or obesity, whereas greater nutrition knowledge was inversely associated with BMI ≥ 25 kg/m^2^. Age showed a non-linear positive association with excess body weight, suggesting that the risk did not increase in a strictly linear manner across the analysed age span. The positive association between higher pHDI scores and BMI ≥ 25 kg/m^2^ also requires cautious interpretation. Multicollinearity diagnostics argue against problematic overlap between pHDI and nHDI, suggesting that this result is unlikely to be a statistical artefact of collinearity. A more plausible explanation is reverse causation or compensatory dietary change: individuals with overweight or obesity may already be attempting to improve their diet by increasing the intake of foods conventionally considered healthier, while still maintaining an overall dietary pattern or energy balance conducive to excess body weight. Because the KomPAN FFQ used in this study captures relative frequency of food-group intake rather than total energy or nutrient intake, it was not possible to determine whether higher pHDI reflected genuinely healthier overall diets or selective dietary modifications undertaken after weight gain. Therefore, this finding should not be interpreted as evidence that pro-healthy foods increase obesity risk, but rather as a cross-sectional signal that recommended-food intake may also reflect behaviour change in response to pre-existing weight concerns [3,25].

Overall, our findings support and extend previous evidence by showing that, in adolescents and young adults, the obesity–depression nexus is embedded in a broader context of incomplete basic nutrition knowledge, variable digital health literacy, unsupervised supplementation, and social media-driven information environments. The coexistence of declarative awareness of the gut–brain axis and psychobiotics with low diet quality, entrenched myths, and poor recognition of somatic symptoms suggests that current educational approaches do not sufficiently translate mechanistic knowledge into every day, actionable decisions. Our findings regarding the underweight subgroup warrant further attention in the context of the gut–brain axis. As suggested by the recent literature, both obesity and undernutrition can lead to intestinal dysbiosis and reduced microbial diversity. Nutrient deficiencies common in undernutrition may alter endocrine and immunological pathways, potentially affecting mental well-being and psychosomatic health. In our study, the high prevalence of supplementation in underweight individuals may reflect an attempt to compensate for perceived deficiencies or to manage body image through non-dietary means [42]. Effective preventive strategies for this age group should therefore combine: (i) strengthening of school-based education in nutrition, food hygiene, and microbiology (including mycotoxins, lipid profiles, and plant-based sources of key nutrients), (ii) systematic training in digital health literacy and critical appraisal of online content, and (iii) closer integration of psychological and dietary support in clinical and public health programmes addressing obesity and mood disorders [13,16,27,28,36,37]. Given that social media reliance in our cohort was linked more clearly to supplementation behaviours than to overall diet quality indices, digital health literacy interventions should explicitly include “product literacy”—skills for evaluating supplement marketing claims, verifying evidence and safety (including contraindications and interactions), and recognising when professional consultation is warranted (Table 8). Consistent with our correlation analysis, higher nutrition knowledge co-occurred with better overall diet quality and higher gut–brain literacy, whereas BMI showed only weak associations with diet indices, underscoring that knowledge-related competencies may be a more consistent correlate of diet quality than body weight itself in this sample (Figure 3).

This study has several limitations. First, its cross-sectional design precludes causal inference regarding the relationships between diet quality, supplementation, health literacy, obesity, and depressive symptoms; observed associations may be bidirectional or confounded by unmeasured variables. Second, all data were self-reported and thus subject to recall and social desirability biases, especially regarding anthropometric measures, supplement use, and mental health-related items; although implausible values were excluded, some misclassification is likely. Third, the purposive online recruitment strategy limits generalisability to all Polish adolescents and young adults, particularly those with limited internet access or from socioeconomically disadvantaged backgrounds. Fourth, depressive symptoms and psychosomatic awareness were assessed using study-specific questionnaire items rather than standardised diagnostic interviews or validated depression scales, constraining the depth of mental health assessment. Finally, diet quality indices were derived from FFQ frequency data and reflect relative consumption patterns rather than precise nutrient intakes, and some measurement error is therefore unavoidable [3]. Additional limitations include the predominance of women in the sample, which may influence estimates of knowledge and health-seeking behaviours, and the absence of detailed socioeconomic and physical activity measures that could further explain variability in BMI and lifestyle-related outcomes.

Despite these limitations, the study provides a comprehensive, psychosomatic perspective on the interplay between diet quality, health literacy, supplementation, and obesity–depression comorbidity in Polish adolescents and young adults. By combining standardised diet quality indices with detailed assessments of nutrition knowledge, psychosomatic awareness, and digital information sources, it helps to define concrete educational and clinical targets for early prevention strategies in this vulnerable life stage [25,27,28,32,36].

## 5. Limitations

Several limitations of this study must be acknowledged. First, the cross-sectional design prevents establishment of causal relationships between the variables and does not allow temporal ordering of the observed associations. Second, the sample was substantially female-dominated, and recruitment was conducted mainly through university mailing lists, institutional channels, and social media. As a result, the study group may overrepresent individuals with relatively high digital engagement, student status, and greater health awareness, which may have influenced both supplementation practices and preferred information pathways. This limits generalisability to all Polish adolescents and young adults. The sex imbalance may also have affected the magnitude of some observed associations, particularly those related to diet quality and BMI. To address the possible impact of sex imbalance, sex-stratified sensitivity analyses were additionally performed for the supplementation model. The association between social media as an information source and supplement use remained positive in both women (OR = 2.21, 95% CI: 1.23–3.99; *p* = 0.0083) and men (OR = 2.57, 95% CI: 1.18–5.61; *p* = 0.0174), suggesting that the main finding was not driven solely by the predominance of women in the sample (Table S10). Third, all anthropometric data were self-reported, and BMI may therefore have been affected by systematic underreporting of weight and overreporting of height, which may have influenced associations observed for BMI ≥ 25 kg/m^2^. Fourth, the use of the KomPAN FFQ provides qualitative frequency data but does not quantify total energy or nutrient intake. Therefore, the dietary indices reflect relative consumption frequencies, and any implications regarding omega-3 fatty acids, iron, B vitamins, or other nutrients should be interpreted as indirect inferences from food-group intake rather than direct assessment of nutrient adequacy. Finally, although the nutrition knowledge scale was piloted and showed acceptable internal consistency, the questionnaire remained author-designed and was not fully validated across different populations.

Future research should therefore use longitudinal designs to clarify the temporal relationships between nutrition knowledge, information sources, dietary behaviours, and supplementation. Mixed-methods approaches may additionally help explain the motivations underlying social media-driven supplementation and the persistence of nutrition misconceptions in young people.

## 6. Conclusions

In this cohort of Polish adolescents and young adults, overall diet quality was generally low to moderate, with limited intake of whole grains, legumes, and fish, and only “sufficient” nutrition knowledge despite declared pro-health attitudes. Social media and online content were commonly reported as major sources of nutrition- and mental health-related information and were particularly associated with supplementation behaviours rather than with overall diet quality indices.

Adolescents tended to present less favourable dietary patterns and a higher prevalence of selected nutrition misconceptions than young adults, particularly regarding vegetarian diets, fats, and marine fish. Awareness of the bidirectional relationship between obesity and depression and of the gut–brain axis appeared to be largely declarative, with limited recognition of somatic symptoms and only superficial understanding of psychobiotics. In addition, misperception of body weight status was relatively common among participants with overweight and obesity.

Taken together, these findings suggest that preventive efforts in this age group may benefit from stronger nutrition education, food safety education, digital health literacy, and more consistent professional guidance regarding supplementation. The observed associations also indicate that male sex, age-related risk patterns, and lower education and nutrition knowledge may represent relevant targets for future prevention-oriented strategies. Given the cross-sectional design, these findings should be interpreted as associative rather than causal.

## Figures and Tables

**Table 1:** Characteristics of the study sample (%).

Variables	Category	(%)
Gender	Women	65.7
	Men	34.3
Place of residence	Urban	46.9
	Village	53.1
Age	≤19	49.4
	20–25	27.2
	>25	23.4
BMI	Underweight	13.2
	Normal weight	62.8
	Overweight	16.5
	Obesity class I	5.9
	Obesity class II	0.4
	Obesity class III	1.3
Education level	Primary	26.8
	Basic vocational	2.5
	Secondary	51.0
	Higher	18.8
	Higher with a doctoral degree	0.8
Type of work	Sedentary work	38.5
	Physically demanding work	13.8
	Shift work	18.4
	Work with high stress intensity	29.3
Marital status	Single	49.4
	In a relationship	49.8
	Widow/widower	0.8

**Table 2:** Descriptive statistics of continuous variables: median [Q1–Q3], min–max, and skewness (overall and by gender).

Variable	Overall: Median [Q1–Q3]	Overall: Min–Max	Overall: Skewness	Women: Median [Q1–Q3]	Women: Min–Max	Women: Skewness	Men: Median [Q1–Q3]	Men: Min–Max	Men: Skewness
Age (years)	20.00 [18.00–25.00]	17.00–30.00	2.14	20.00 [18.00–26.00]	17.00–30.00	1.92	19.50 [18.00–23.00]	17.00–30.00	2.47
BMI (kg/m^2^)	22.05 [19.95–24.84]	16.15–46.30	1.54	21.22 [19.47–24.14]	16.18–46.30	2.06	23.55 [21.50–26.23]	16.15–38.82	0.73
pHDI (pkt)	20.60 [12.68–31.30]	2.40–65.00	0.81	20.80 [14.40–31.00]	2.40–60.30	0.82	19.45 [11.30–31.70]	3.10–65.00	0.79
nHDI (pkt)	15.86 [10.57–22.29]	0.64–94.64	2.16	14.36 [9.29–20.36]	0.64–58.93	1.31	19.21 [14.86–24.57]	5.00–94.64	3.22
DQI (pkt)	5.06 [−4.72–16.49]	−83.34–50.63	−0.37	6.37 [−1.47–17.03]	−29.64–48.60	0.22	0.54 [−8.66–14.86]	−83.34–50.63	−0.77
Knowledge score (points)	11.00 [9.00–14.00]	0.00–25.00	−0.02	11.00 [10.00–14.00]	0.00–25.00	0.27	11.00 [8.00–14.00]	0.00–20.00	−0.28
Knowledge (%)	44.00 [36.00–56.00]	0.00–92.00	−0.02	44.00 [40.00–56.00]	0.00–92.00	0.27	44.00 [32.00–56.00]	0.00–80.00	−0.28

**Table 3:** Categorical variables by sex (%): chi-square/Fisher tests and Cramér’s V.

Variable	Category	Women (%)	Men (%)	Chi^2^	Fisher *p*	Cramer’s V
Place of residence	Urban	44.6%	51.2%	0.199	0.177	0.059
	Village	55.4%	48.8%			
Age	≤19	49.0%	50.0%	0.266		0.074
	20–25	25.5%	30.5%			
	>25	25.5%	19.5%			
BMI category	Underweight	16.6%	6.7%	<0.001		0.248
	Normal weight	65.0%	58.5%			
	Overweight	12.1%	25.0%			
	Obesity class I	4.5%	8.5%			
	Obesity class II	0.0%	1.2%			
	Obesity class III	1.9%	0.0%			
Education level	Primary	28.0%	24.4%	0.007		0.171
	Basic vocational	2.5%	2.4%			
	Secondary	45.9%	61.0%			
	Higher	22.9%	11.0%			
	Higher with doctoral degree	0.6%	1.2%			
Type of work	Sedentary work	44.6%	26.8%	<0.001		0.288
	Physically demanding work	7.0%	26.8%			
	Shift work	19.1%	17.1%			
	Work with high stress intensity	29.3%	29.3%			
Marital status	Single	42.0%	63.4%	<0.001		0.209
	In a relationship	56.7%	36.6%			
	Widow/widower	1.3%	0.0%			

**Table 4:** Comparison of continuous variables by sex: Mann–Whitney test with effect size r and Cliff’s delta.

Variable	U	*p*	r (95% CI)	Cliff’s Delta (95% CI)
Age (years)	25,655	0.9479	0.003 (0.001–0.104)	−0.004 (−0.110–0.106)
BMI (kg/m^2^)	17,573	<0.0001	0.261 (0.176–0.344)	−0.318 (−0.418–−0.224)
pHDI (pkt)	26,982	0.3896	0.039 (0.002–0.138)	0.048 (−0.070–0.161)
nHDI (pkt)	17,346	<0.0001	0.268 (0.191–0.343)	−0.326 (−0.421–−0.227)
DQI (pkt)	30,596	0.0007	0.155 (0.061–0.246)	0.188 (0.071–0.310)
Knowledge (%)	28,951	0.0249	0.102 (0.019–0.188)	0.124 (0.018–0.229)

Cliff’s delta is reported for women vs. men (negative values indicate lower values in women).

**Table 5:** Associations between diet quality profiles (pHDI-10 × nHDI-14) and sociodemographic factors, BMI, and nutrition knowledge.

Variable	Category/Statistic	Total	L_DQI	M_DQI	U_DQI	*p*-Value	Effect Size
DQI profile	L_DQI	7.1%					
	M_DQI	70.7%					
	U_DQI	22.2%					
Sex	Woman	65.7%	47.1%	68.0%	64.2%	0.0455	Cramér’s V = 0.114
	Men	34.3%	52.9%	32.0%	35.8%		
Place of residence	Urban	46.9%	23.5%	46.2%	56.6%	0.0031	Cramér’s V = 0.155
	Village	53.1%	76.5%	53.8%	43.4%		
Age group (years)	≤19	49.4%	52.9%	50.3%	45.3%	0.2391	Cramér’s V = 0.076
	20–25	27.2%	23.5%	24.9%	35.8%		
	>25	23.4%	23.5%	24.9%	18.9%		
Education	<Higher	80.3%	88.2%	81.7%	73.6%	0.0919	Cramér’s V = 0.100
	Higher	19.7%	11.8%	18.3%	26.4%		
BMI category	Underweight	13.2%	17.6%	13.9%	9.4%	0.3805	Cramér’s V = 0.106
	Normal weight	62.8%	58.8%	65.1%	56.6%		
	Overweight	16.5%	17.6%	14.5%	22.6%		
	Obesity class I	5.9%	5.9%	4.7%	9.4%		
	Obesity class II	0.4%	0.0%	0.6%	0.0%		
	Obesity class III	1.3%	0.0%	1.2%	1.9%		
Nutrition knowledge level (KomPAN)	Insufficient (0–8)	20.1%	23.5%	23.1%	9.4%	0.0182	Cramér’s V = 0.112
	Sufficient (9–16)	68.2%	58.8%	66.9%	75.5%		
	Good (17–25)	11.7%	17.6%	10.1%	15.1%		
Knowledge profile (KomPAN proposal I)	A: >50% correct answers	36.0%	47.1%	29.0%	54.7%	0.0001	Cramér’s V = 0.170
	B: >50% incorrect answers	2.1%	0.0%	2.4%	1.9%		
	C: >50% “difficult to say” answers	15.9%	17.6%	18.3%	7.5%		
	D: others	46.0%	35.3%	50.3%	35.8%		
Age (years)	Median [Q1–Q3]	20.00 [18.00–25.00]	19.00 [18.00–24.00]	19.00 [18.00–25.00]	21.00 [18.00–24.00]	0.4022	ε^2^ = −0.000
BMI (kg/m^2^)	Median [Q1–Q3]	22.05 [19.95–24.84]	21.33 [19.47–22.86]	21.83 [19.81–24.42]	23.55 [20.68–26.35]	0.0042	ε^2^ = 0.019
pHDI-10 (points)	Median [Q1–Q3]	20.60 [12.68–31.30]	18.00 [10.00–24.00]	17.70 [11.60–23.80]	42.50 [37.00–48.90]	<0.0001	ε^2^ = 0.516
nHDI-14 (points)	Median [Q1–Q3]	15.86 [10.57–22.29]	36.14 [34.57–43.50]	14.93 [9.93–19.86]	16.50 [11.64–22.64]	<0.0001	ε^2^ = 0.197
DQI (points)	Median [Q1–Q3]	5.06 [−4.72–16.49]	−24.26 [−27.36–−12.66]	2.53 [−4.40–10.84]	25.16 [18.57–31.86]	<0.0001	ε^2^ = 0.473
Knowledge score (0–25)	Median [Q1–Q3]	11.00 [9.00–14.00]	10.00 [9.00–14.00]	11.00 [9.00–13.00]	13.00 [10.00–15.00]	<0.0001	ε^2^ = 0.033

Abbreviations: pHDI—pro-healthy diet index; nHDI—non-healthy diet index; DQI—diet quality index; ε^2^—epsilon-squared effect size; Cramér’s V—effect size for χ^2^. DQI profiles were defined from pHDI_kat and nHDI_kat categories in this sample.

**Table 6:** Diet quality index profiles with the proportion of study participants characterized by a given profile.

Profile Number	Type of Indicator	%
1	Low pHDI-10 and low nHDI-14	70.7
2	Low pHDI-10 and moderate nHDI-14	6.7
3	Low pHDI-10 and high nHDI-14	0.4
4	Moderate pHDI-10 and low nHDI-14	20.1
5	Moderate pHDI-10 and moderate nHDI-14	2.1

**Table 7:** Characteristics of dietary-behaviour clusters identified by exploratory cluster analysis.

Cluster	N (%)	pHDI-10, Median	nHDI-14, Median	DQI, Median	Knowledge Score, Median	Gut–Brain Literacy, Median	Underweight, %	BMI ≥ 25, %	Any Supplementation, %
Higher-pro-healthy/relatively regular pattern	150 (31.4)	33.5	15.9	17.9	11	3	5.3	32.0	80.0
Lower-unhealthy/lower-intensity pattern	140 (29.3)	17.5	9.9	6.3	11	3	16.4	19.3	88.6
Unhealthy–irregular pattern	188 (39.3)	16.8	21.6	−5.1	10	2	17.0	21.3	71.3

**Notes:** Overall, between-cluster differences were significant for nutrition knowledge (*p* < 0.001), gut–brain literacy (*p* < 0.001), BMI category (*p* < 0.001), supplementation (*p* < 0.001), sex (*p* = 0.005), and age group (*p* = 0.008), but not for social media as a source of information (*p* = 0.982).

**Table 8:** Associations of social media as an information source with supplementation, diet quality indices and knowledge.

**A. Supplement use by social media information source (chi-square *p* = 0.0049; Cramer’s V = 0.129)**				
**Measure**	**SM = No**		**SM = Yes**	
Supplement use (yes or sometimes)	150 (72.8%)		228 (83.8%)	
No supplement use	56 (27.2%)		44 (16.2%)	
Total (within SM group)	206 (100%)		272 (100%)	
**B. Diet quality indices and nutrition knowledge by social media information source (Mann–Whitney)**				
**Outcome**	**Median (SM = Yes)**	**Median (SM = No)**	***p***	**Rank-biserial r**
pHDI-10	20.3	20.6	0.9555	−0.003
nHDI-14	15.1071	16.1429	0.1604	0.075
DQI	4.9286	5.0714	0.6592	−0.0236
Nutrition knowledge score (0–25)	11.0	11.0	0.0335	0.113
**C. Multivariable logistic regression: predictors of supplement use (yes/sometimes vs. no)**				
**Predictor**	**OR (95% CI)**		***p***	
Male sex (ref: female)	1.01 (0.63–1.64)		0.9554	
Age (per 1 year)	1.03 (0.99–1.06)		0.1255	
Tertiary education (ref: lower)	0.96 (0.45–2.04)		0.9221	
Nutrition knowledge (per 1 point)	1.09 (1.02–1.16)		0.0111	
DQI (per 1 point)	1.00 (0.99–1.02)		0.8025	
Social media as an information source (yes vs. no)	2.29 (1.43–3.64)		0.0005	

Notes: SM indicates selecting social media as a source of information about diet and supplements. In Block A, percentages are within SM groups. Block B reports medians and Mann–Whitney *p*-values. Block C reports adjusted odds ratios (ORs) with 95% confidence intervals.

**Table 9:** Logistic regression: predictors of BMI ≥ 25 kg/m^2^—OR and 95% CI.

Predictor	OR (95% CI)	*p*
Gender: male (ref. female)	2.46 (1.46–4.15)	0.0008
Place of residence: rural (ref. urban)	0.80 (0.49–1.28)	0.3485
Education: higher (ref. <higher)	0.21 (0.09–0.45)	<0.001
Type of work: sedentary (ref. other)	0.68 (0.40–1.14)	0.1433
Marital status: in a relationship (ref. not in a relationship)	1.38 (0.82–2.33)	0.2274
Age (per 1 year)	1.25 (1.17–1.34)	<0.001
Age^2^ (quadratic term)	0.99 (0.99–0.99)	<0.001
Knowledge score (per 1 point)	0.92 (0.86–0.99)	0.0215
pHDI (per 1 point)	1.04 (1.02–1.06)	<0.001
nHDI (per 1 point)	0.98 (0.96–1.01)	0.2229

Note: Age was entered as centred linear and quadratic terms because assessment of linearity of the logit indicated a non-linear age effect. The final model showed acceptable calibration (Hosmer–Lemeshow χ^2^ = 11.84, *p* = 0.159), acceptable discrimination (AUC = 0.777), and Nagelkerke’s R^2^ = 0.268.

## Data Availability

The anonymised data underlying this study are available from the corresponding author upon reasonable request. Public deposition was not implemented because the dataset contains detailed health- and behaviour-related responses from adolescents and young adults, and additional ethical safeguards were considered necessary to minimise re-identification risk.

## Supplementary Materials

The following supporting information can be downloaded at https://www.mdpi.com/article/10.3390/nu18091363/s1. Table S1. Agreement between BMI category (WHO) and self-perceived body weight status among respondents (N = 478); Table S2. Supplementation practice and sources of information (A–D); Table S3. Exploratory multivariable logistic regression models; Table S4. Coding of open-ended responses (N = 478); Table S5. Gut–brain literacy indicators in the study sample (N = 478): awareness, beliefs, and operational knowledge regarding probiotics/psychobiotics and microbiota-related concepts; Table S6. Comparison of FFQ food frequency responses between adolescents and young adults; Table S7. Nutrition knowledge statements (true/false/no opinion) in adolescents and young adults; Table S8. Health beliefs and self-rated mental well-being by age group; Table S9. Depression literacy and symptom recognition in adolescents and young adults; Table S10. Sex-stratified sensitivity analyses for the supplementation model; Table S11. Self-reported chronic conditions in the study sample.
