# Supplementary material for: Diet Quality, Nutrition Knowledge, and Social Media-Driven Supplement Use Among Polish Adolescents and Young Adults: A Cross-Sectional Study
Source: Nutrients. 2026 Apr 25;18(9):1363. doi: 10.3390/nu18091363 (PMC13165024; doi:10.3390/nu18091363)
Supplement: Supplementary file 1 [file nutrients-18-01363-s001.zip › nutrients-4242833-supplementary.pdf]

nutrients

MDPI

Article- Suplementary material

Diet Quality, Nutrition Knowledge, and Social Media-Driven Supplement Use Among Polish Adolescents and Young Adults: A Cross-Sectional Study

Klaudia Sochacka[1], Agata Kotowska[2], Sabina Lachowicz-Wiśniewska[3,4,*]

1 Doctoral School, The Faculty of Medicine and Health Science, University of Kalisz, W. Bogusławskiego 2, 62-800 Kalisz, Poland; szd.5.2023@uniwersytetkaliski.edu.pl
2 Institute of Sociology, University of Rzeszów, 35-959 Rzeszów, Poland, akotowska@ur.edu.pl;
3 Department of Nutrition and Food, The Faculty of Medicine and Health Science, University of Kalisz, W. Bogusławskiego 2, 62-800 Kalisz, Poland;
4 Department of Biotechnology and Food Analysis, Wroclaw University of Economics and Business, 53-345 Wroclaw, Poland;
* Correspondence: s.lachowicz-wisniewska@uniwersytetkaliski.edu.pl

Abstract

Diet quality, nutrition knowledge, and psychosomatic literacy—defined as the understanding of the interactions between diet, gut microbiota, and mental well-being—may shape weight-related behaviours in youth. This study used a cross-sectional design to integrate these domains with digital information pathways in Central–Eastern Europe. This study assessed diet quality, nutrition, and psychosomatic knowledge, supplement use, and health-information sources among Polish adolescents and young adults, with emphasis on age-related differences and the role of social media. A cross-sectional, anonymous online survey (October 2025–January 2026) was conducted in Poland (final analytical sample: n = 478; adolescents 15–19 years vs young adults 20–30 years). Of 591 individuals who accessed the survey, 478 were included in the final analytical sample. Diet quality was estimated from FFQ data using KomPAN-derived indices (pHDI-10, nHDI-14, DQI). Nutrition knowledge (0–25 points), psychosomatic/gut–brain indicators, supplementation, and information sources were analysed using χ^2/Fisher tests and Mann–Whitney U tests with effect sizes. The primary outcomes measured were dietary supplement use and excess body weight (BMI ≥25 kg/m^2). Multivariable logistic regression examined predictors of supplement use and BMI ≥ 25 kg/m^2. Overall diet quality was low-to-moderate, with limited intake of whole grains, legumes, and fish, and common nutrition misconceptions. Social media was the most frequently indicated source of diet/supplement information and was independently associated with more frequent supplement use (OR = 2.29; 95% CI: 1.43–3.64). Adolescents reported lower whole-grain intake and more misconceptions than young adults. Predictors of BMI ≥ 25 kg/m^2 included male sex (OR = 2.46; 95% CI: 1.46–4.15), lower education, and lower nutrition knowledge, while age showed a non-linear positive association with excess body weight. Polish adolescents and young adults show gaps between declared pro-health attitudes and actual diet quality/competencies. Social media reliance appears particularly linked to product-oriented behaviours (supplementation). Prevention should strengthen nutrition and food-safety education, digital health literacy, and professional guidance on supplementation, especially in adolescents. Our findings suggest that social media is a primary driver for dietary supplementation among Polish y, more so than objective nutrition knowledge. While diet quality is linked

Academic Editor: Firstname Lastname

Received: date
Revised: date
Accepted: date
Published: date

Copyright: © 2026 by the authors. Submitted for possible open access publication under the terms and conditions of the Creative Commons Attribution (CC BY) license.

to weight status, the relationship is complex. These results may inform future public health interventions targeting digital health literacy to promote balanced nutrition and safe supplementation practices.

Tabela S1. Agreement between BMI category (WHO) and self-perceived body weight status among respondents (n = 478)*

BMI	n in the BMI group	Hard to say	Perceives normal	Perceives obese	Perceives overweight/too high	Perceives too low
Underweight	63	4 (6.3%)	22 (34.9%)	0 (0.0%)	5 (7.9%)	32 (50.8%)
Normal weight	300	18 (6.0%)	210 (70.0%)	2 (0.7%)	44 (14.7%)	26 (8.7%)
Overweight	79	6 (7.6%)	24 (30.4%)	4 (5.1%)	45 (57.0%)	0 (0.0%)
Obesity (I–III)	36	2 (5.6%)	2 (5.6%)	20 (55.6%)	12 (33.3%)	0 (0.0%)

* Chi-square *p*-value <0.001, Cramér's V 0.513, N=478

Table S2. Supplementation practice and sources of information (A–D).

A. Supplement use (vitamins/other supplements) by age group

Category	Total	Adolescents	Young adults
Yes	320 (66.9%)	146 (61.9%)	174 (71.9%)
Sometimes	58 (12.1%)	40 (16.9%)	18 (7.4%)
No	100 (20.9%)	50 (21.2%)	50 (20.7%)

B. Perceived knowledge on supplementation justification by age group

Category	Total	Adolescents	Young adults
Yes	118 (24.7%)	46 (19.5%)	72 (29.8%)
I think so	250 (52.3%)	124 (52.5%)	126 (52.1%)
No	110 (23.0%)	66 (28.0%)	44 (18.2%)

C. Recommendation patterns among supplement users (n = 378)

Recommendation pattern	n (%)
Self only	264 (69.8%)
Physician only	68 (18.0%)
Dietitian only	24 (6.3%)
Self + physician	16 (4.2%)
Self + physician + dietitian	4 (1.1%)
Physician + dietitian	2 (0.5%)

D. Sources of information about diet and supplements (multiple responses allowed)

Source (multiple responses allowed)	Total n (%)	Adolescents n (%)	Young adults n (%)	p (χ^2)	Cramér's V
Social media	272 (56.9%)	142 (60.2%)	130 (53.7%)	0.183	0.061
Online articles	206 (43.1%)	74 (31.4%)	132 (54.5%)	<0.001	0.230
Family	224 (46.9%)	142 (60.2%)	82 (33.9%)	<0.001	0.259
Educational media programmes (guides, popular science, lifestyle programmes)	178 (37.2%)	82 (34.7%)	96 (39.7%)	0.308	0.047
Friends/peers	130 (27.2%)	72 (30.5%)	58 (24.0%)	0.133	0.069

School-based educational programmes (e.g., "5 portions of vegetables, fruit or juice")	46 (9.6%)	28 (11.9%)	18 (7.4%)	0.137	0.068
Public health campaigns (e.g., "Do not serve yourself dis-ease!", "Plan a long life")	36 (7.5%)	10 (4.2%)	26 (10.7%)	0.012	0.115

Notes: Values are presented as n (%). In Block D, multiple responses were allowed; percentages refer to the total number of respondents in each age group and in the total sample. p-values are from χ^2 tests comparing adolescents vs young adults for each source.

Table S3. Exploratory multivariable logistic regression models.

**A. Predictors of low diet quality (L_DQI) (N = 478; cases n = 34)**		
**Predictor**	**OR (95% CI)**	**p**
Male sex (ref: female)	2.34 (1.14–4.80)	0.0207
Age (per 1 year)	1.01 (0.96–1.05)	0.7013
Tertiary education (ref: lower)	0.45 (0.14–1.48)	0.1899
Nutrition knowledge (0–25; per 1 point)	1.08 (0.98–1.19)	0.1159
Social media as information source (yes vs no)	1.03 (0.49–2.15)	0.9389
Supplement use (yes/sometimes vs no)	0.42 (0.19–0.92)	0.0296
**B. Predictors of high gut–brain literacy (N = 478; high literacy n = 274)**		
**Predictor**	**OR (95% CI)**	**p**
Male sex (ref: female)	0.63 (0.42–0.96)	0.0318
Age (per 1 year)	0.97 (0.94–0.99)	0.0128
Tertiary education (ref: lower)	1.50 (0.81–2.77)	0.1944
Nutrition knowledge (0–25; per 1 point)	1.24 (1.16–1.32)	<0.001
Social media as information source (yes vs no)	1.24 (0.82–1.86)	0.3026

Notes: L_DQI indicates profiles with low pro-healthy diet index and moderate/high non-healthy diet index. High gut–brain literacy was defined as a score ≥3 on the 0–4 gut–brain literacy indicator (diet–microbiota knowledge, knowledge of probiotics/psychobiotics, belief in mood effects, and knowledge of typical psychobiotic strains). OR denotes adjusted odds ratio; CI, confidence interval. Models are exploratory.

Table S4. Coding of open-ended responses (N = 478).

**A. Potential nutrient deficiencies reported by respondents (coded categories)**	
**Category**	**n (%)**
Iron	196 (41.0%)
Vitamin D	192 (40.2%)
Vitamin B12	130 (27.2%)
Magnesium	8 (1.7%)
Zinc	4 (0.8%)
Protein	4 (0.8%)
Vitamin C	2 (0.4%)
Omega-3	2 (0.4%)
**B. Reported strategies to reduce simple carbohydrate intake (coded categories)**	
**Category**	**n (%)**
Choose products with reduced simple sugars	240 (50.2%)
Avoid products with hidden sugar	224 (46.9%)
Limit added sugar	130 (27.2%)

Cook/bake without fat (selected option)	108 (22.6%)
**C. Herbs/supplements perceived to support depression (coded categories)**	
**Category**	**n (%)**
Lemon balm	29 (6.1%)
Vitamin D	21 (4.4%)
Ashwagandha	20 (4.2%)
St John's wort	16 (3.3%)
Omega-3	7 (1.5%)
Valerian	4 (0.8%)
Magnesium	4 (0.8%)
**D. Herbs/supplements perceived to support obesity management (coded categories)**	
**Category**	**n (%)**
Green tea	12 (2.5%)
White mulberry	7 (1.5%)
Chromium	3 (0.6%)
Probiotics	2 (0.4%)
Berberine	1 (0.2%)
Caffeine/thermogenics	1 (0.2%)
**E. Foods perceived as rich in live bacterial cultures (coded categories)**	
**Category**	**n (%)**
Yogurt	162 (33.9%)
Kefir	137 (28.7%)
Sauerkraut/pickles	54 (11.3%)
Buttermilk/fermented milk	36 (7.5%)
Kimchi	4 (0.8%)
Kombucha	2 (0.4%)
Sourdough	2 (0.4%)

Notes: Categories were derived from coding of open-ended responses. Categories are not mutually exclusive; a single response could be coded into multiple categories. Percentages refer to the total sample (N = 478).

Table S5. Gut–brain literacy indicators in the study sample (N = 478): awareness, beliefs, and operational knowledge regarding probiotics/psychobiotics and microbiota-related concepts.

Item	Yes	No	Don't know/No knowledge
Awareness: depression can influence obesity symptoms	390 (81.6%)	24 (5.0%)	64 (13.4%)
Knowledge: diet can influence gut microbiota composition	392 (82.0%)	10 (2.1%)	76 (15.9%)
Self-declared knowledge: what probiotics/psychobiotics are	376 (78.7%)	102 (21.3%)	—
Belief: probiotics/psychobiotics can improve mood	346 (72.4%)	132 (27.6%)	0 (0.0%)
Operational knowledge: knows typical psychobiotic strains	66 (13.8%)	90 (18.8%)	322 (67.4%)
Food knowledge: bio-yogurts contain beneficial gut bacteria	308 (64.4%)	26 (5.4%)	144 (30.1%)

Table S6. Comparison of FFQ food-frequency responses between adolescents and young adults.

Food group	Frequency	Total n (%)	Adolescents n (%)	Young adults n (%)	p-value	Cramer's V

Wholemeal bread	Never	100 (20.9%)	46 (19.5%)	54 (22.3%)	0.6791	0.081
	1-3 times per month	130 (27.2%)	60 (25.4%)	70 (28.9%)		
	Once per week	52 (10.9%)	26 (11.0%)	26 (10.7%)		
	Several times per week	126 (26.4%)	66 (28.0%)	60 (24.8%)		
	Once per day	48 (10.0%)	28 (11.9%)	20 (8.3%)		
	Several times per day	22 (4.6%)	10 (4.2%)	12 (5.0%)		
Whole-grain groats/oats/whole-grain pasta	Never	48 (10.0%)	20 (8.5%)	28 (11.6%)	0.2376	0.119
	1-3 times per month	170 (35.6%)	82 (34.7%)	88 (36.4%)		
	Once per week	70 (14.6%)	42 (17.8%)	28 (11.6%)		
	Several times per week	148 (31.0%)	76 (32.2%)	72 (29.8%)		
	Once per day	32 (6.7%)	12 (5.1%)	20 (8.3%)		
	Several times per day	10 (2.1%)	4 (1.7%)	6 (2.5%)		
Milk (incl. flavoured milk/cocoa/coffee with milk)	Never	48 (10.0%)	16 (6.8%)	32 (13.2%)	0.0309	0.160
	1-3 times per month	78 (16.3%)	36 (15.3%)	42 (17.4%)		
	Once per week	54 (11.3%)	34 (14.4%)	20 (8.3%)		
	Several times per week	142 (29.7%)	78 (33.1%)	64 (26.4%)		
	Once per day	92 (19.2%)	40 (16.9%)	52 (21.5%)		
	Several times per day	64 (13.4%)	32 (13.6%)	32 (13.2%)		
Fermented dairy (yogurt/kefir)	Never	32 (6.7%)	16 (6.8%)	16 (6.6%)	0.0566	0.150
	1-3 times per month	124 (25.9%)	62 (26.3%)	62 (25.6%)		
	Once per week	64 (13.4%)	42 (17.8%)	22 (9.1%)		
	Several times per week	154 (32.2%)	68 (28.8%)	86 (35.5%)		
	Once per day	80 (16.7%)	34 (14.4%)	46 (19.0%)		
	Several times per day	24 (5.0%)	14 (5.9%)	10 (4.1%)		
White meat (chicken/turkey/rabbit)	Never	12 (2.5%)	6 (2.5%)	6 (2.5%)	0.0165	0.170
	1-3 times per month	26 (5.4%)	6 (2.5%)	20 (8.3%)		
	Once per week	50 (10.5%)	18 (7.6%)	32 (13.2%)		
	Several times per week	268 (56.1%)	138 (58.5%)	130 (53.7%)		
	Once per day	110 (23.0%)	60 (25.4%)	50 (20.7%)		
	Several times per day	12 (2.5%)	8 (3.4%)	4 (1.7%)		
Fish	Never	60 (12.6%)	36 (15.3%)	24 (9.9%)	0.0363	0.158
	1-3 times per month	264 (55.2%)	128 (54.2%)	136 (56.2%)		
	Once per week	92 (19.2%)	36 (15.3%)	56 (23.1%)		
	Several times per week	52 (10.9%)	28 (11.9%)	24 (9.9%)		
	Once per day	6 (1.3%)	4 (1.7%)	2 (0.8%)		
	Several times per day	4 (0.8%)	4 (1.7%)	0 (0.0%)		
Legumes (beans/peas/soy/lentils)	Never	94 (19.7%)	50 (21.2%)	44 (18.2%)	0.5678	0.090
	1-3 times per month	240 (50.2%)	110 (46.6%)	130 (53.7%)		
	Once per week	62 (13.0%)	36 (15.3%)	26 (10.7%)		
	Several times per week	64 (13.4%)	32 (13.6%)	32 (13.2%)		
	Once per day	14 (2.9%)	6 (2.5%)	8 (3.3%)		
	Several times per day	4 (0.8%)	2 (0.8%)	2 (0.8%)		
Fruit	Never	0 (0.0%)	0 (0.0%)	0 (0.0%)	0.0235	0.154

	1-3 times per month	44 (9.2%)	22 (9.3%)	22 (9.1%)		
	Once per week	64 (13.4%)	28 (11.9%)	36 (14.9%)		
	Several times per week	198 (41.4%)	112 (47.5%)	86 (35.5%)		
	Once per day	98 (20.5%)	36 (15.3%)	62 (25.6%)		
	Several times per day	74 (15.5%)	38 (16.1%)	36 (14.9%)		
Vegetables	Never	2 (0.4%)	2 (0.8%)	0 (0.0%)	0.2907	0.114
	1-3 times per month	18 (3.8%)	12 (5.1%)	6 (2.5%)		
	Once per week	34 (7.1%)	16 (6.8%)	18 (7.4%)		
	Several times per week	174 (36.4%)	88 (37.3%)	86 (35.5%)		
	Once per day	126 (26.4%)	64 (27.1%)	62 (25.6%)		
	Several times per day	124 (25.9%)	54 (22.9%)	70 (28.9%)		
White/refined bread and rolls	Never	18 (3.8%)	8 (3.4%)	10 (4.1%)	0.0415	0.155
	1-3 times per month	42 (8.8%)	12 (5.1%)	30 (12.4%)		
	Once per week	54 (11.3%)	24 (10.2%)	30 (12.4%)		
	Several times per week	148 (31.0%)	84 (35.6%)	64 (26.4%)		
	Once per day	136 (28.5%)	70 (29.7%)	66 (27.3%)		
	Several times per day	80 (16.7%)	38 (16.1%)	42 (17.4%)		
Refined grains (white rice/regular pasta/fine groats)	Never	16 (3.3%)	8 (3.4%)	8 (3.3%)	0.0366	0.158
	1-3 times per month	88 (18.4%)	30 (12.7%)	58 (24.0%)		
	Once per week	94 (19.7%)	52 (22.0%)	42 (17.4%)		
	Several times per week	210 (43.9%)	106 (44.9%)	104 (43.0%)		
	Once per day	58 (12.1%)	32 (13.6%)	26 (10.7%)		
	Several times per day	12 (2.5%)	8 (3.4%)	4 (1.7%)		
Fast food (fries/burgers/pizza/hot dogs)	Never	50 (10.5%)	14 (5.9%)	36 (14.9%)	0.0024	0.196
	1-3 times per month	310 (64.9%)	164 (69.5%)	146 (60.3%)		
	Once per week	58 (12.1%)	24 (10.2%)	34 (14.0%)		
	Several times per week	54 (11.3%)	28 (11.9%)	26 (10.7%)		
	Once per day	4 (0.8%)	4 (1.7%)	0 (0.0%)		
	Several times per day	2 (0.4%)	2 (0.8%)	0 (0.0%)		
Butter	Never	52 (10.9%)	26 (11.0%)	26 (10.7%)	0.1451	0.131
	1-3 times per month	50 (10.5%)	22 (9.3%)	28 (11.6%)		
	Once per week	40 (8.4%)	16 (6.8%)	24 (9.9%)		
	Several times per week	132 (27.6%)	76 (32.2%)	56 (23.1%)		
	Once per day	134 (28.0%)	58 (24.6%)	76 (31.4%)		
	Several times per day	70 (14.6%)	38 (16.1%)	32 (13.2%)		
Lard	Never	334 (69.9%)	170 (72.0%)	164 (67.8%)	0.0016	0.201
	1-3 times per month	96 (20.1%)	34 (14.4%)	62 (25.6%)		
	Once per week	20 (4.2%)	10 (4.2%)	10 (4.1%)		
	Several times per week	20 (4.2%)	16 (6.8%)	4 (1.7%)		
	Once per day	4 (0.8%)	2 (0.8%)	2 (0.8%)		
	Several times per day	4 (0.8%)	4 (1.7%)	0 (0.0%)		
Sweets/confectionery	Never	12 (2.5%)	8 (3.4%)	4 (1.7%)	0.0033	0.192
	1-3 times per month	84 (17.6%)	30 (12.7%)	54 (22.3%)		

	Once per week	88 (18.4%)	44 (18.6%)	44 (18.2%)		
	Several times per week	158 (33.1%)	70 (29.7%)	88 (36.4%)		
	Once per day	74 (15.5%)	46 (19.5%)	28 (11.6%)		
	Several times per day	62 (13.0%)	38 (16.1%)	24 (9.9%)		
Sugar-sweetened beverages	Never	96 (20.1%)	36 (15.3%)	60 (24.8%)	0.0005	0.215
	1-3 times per month	150 (31.4%)	64 (27.1%)	86 (35.5%)		
	Once per week	74 (15.5%)	50 (21.2%)	24 (9.9%)		
	Several times per week	114 (23.8%)	58 (24.6%)	56 (23.1%)		
	Once per day	24 (5.0%)	14 (5.9%)	10 (4.1%)		
	Several times per day	20 (4.2%)	14 (5.9%)	6 (2.5%)		
Energy drinks	Never	212 (44.4%)	82 (34.7%)	130 (53.7%)	<0.0001	0.245
	1-3 times per month	106 (22.2%)	58 (24.6%)	48 (19.8%)		
	Once per week	68 (14.2%)	40 (16.9%)	28 (11.6%)		
	Several times per week	52 (10.9%)	38 (16.1%)	14 (5.8%)		
	Once per day	24 (5.0%)	8 (3.4%)	16 (6.6%)		
	Several times per day	16 (3.3%)	10 (4.2%)	6 (2.5%)		
Alcoholic beverages	Never	196 (41.0%)	96 (40.7%)	100 (41.3%)	0.6913	0.080
	1-3 times per month	210 (43.9%)	106 (44.9%)	104 (43.0%)		
	Once per week	44 (9.2%)	20 (8.5%)	24 (9.9%)		
	Several times per week	20 (4.2%)	10 (4.2%)	10 (4.1%)		
	Once per day	2 (0.4%)	0 (0.0%)	2 (0.8%)		
	Several times per day	6 (1.3%)	4 (1.7%)	2 (0.8%)		

Adolescents: n = 236 (age <= 19 years); Young adults: n = 242 (age >= 20 years). Values are n (%). p-values are from chi-square tests; effect size is Cramer's V.

Table S7. Nutrition knowledge statements (True/False/No opinion) in adolescents and young adults.

Statement	Response	Total n (%)	Adolescents n (%)	Young adults n (%)	p-value	Cramer's V
Cereal products are sufficient once per day	True	230 (48.1%)	114 (48.3%)	116 (47.9%)	<0.0001	0.223
	False	86 (18.0%)	24 (10.2%)	62 (25.6%)		
	No opinion	162 (33.9%)	98 (41.5%)	64 (26.4%)		
Only children and adolescents should consume milk	True	64 (13.4%)	34 (14.4%)	30 (12.4%)	0.0502	0.112
	False	380 (79.5%)	192 (81.4%)	188 (77.7%)		
	No opinion	34 (7.1%)	10 (4.2%)	24 (9.9%)		
Fruit and/or vegetables should be eaten at every meal	True	382 (79.9%)	184 (78.0%)	198 (81.8%)	0.0447	0.114
	False	58 (12.1%)	26 (11.0%)	32 (13.2%)		
	No opinion	38 (7.9%)	26 (11.0%)	12 (5.0%)		
Eating mouldy bread may cause Salmonella infection	True	222 (46.4%)	130 (55.1%)	92 (38.0%)	0.0005	0.179
	False	110 (23.0%)	50 (21.2%)	60 (24.8%)		
	No opinion	146 (30.5%)	56 (23.7%)	90 (37.2%)		
High salt intake protects against hypertension	True	36 (7.5%)	12 (5.1%)	24 (9.9%)	0.0004	0.182
	False	380 (79.5%)	180 (76.3%)	200 (82.6%)		
	No opinion	62 (13.0%)	44 (18.6%)	18 (7.4%)		

Limiting fatty dishes helps prevent cardiovascular diseases	True	410 (85.8%)	198 (83.9%)	212 (87.6%)	0.5104	0.053
	False	18 (3.8%)	10 (4.2%)	8 (3.3%)		
	No opinion	50 (10.5%)	28 (11.9%)	22 (9.1%)		
Frequent consumption of fatty sea fish accelerates atherosclerosis	True	78 (16.3%)	50 (21.2%)	28 (11.6%)	<0.0001	0.244
	False	186 (38.9%)	64 (27.1%)	122 (50.4%)		
	No opinion	214 (44.8%)	122 (51.7%)	92 (38.0%)		
Eating grilled meat promotes cancer development	True	226 (47.3%)	120 (50.8%)	106 (43.8%)	0.0175	0.130
	False	80 (16.7%)	28 (11.9%)	52 (21.5%)		
	No opinion	172 (36.0%)	88 (37.3%)	84 (34.7%)		
Vegetarian diets inevitably increase anaemia risk	True	316 (66.1%)	156 (66.1%)	160 (66.1%)	0.3142	0.070
	False	58 (12.1%)	24 (10.2%)	34 (14.0%)		
	No opinion	104 (21.8%)	56 (23.7%)	48 (19.8%)		
Bio-yoghurts contain beneficial gut bacteria	True	308 (64.4%)	154 (65.3%)	154 (63.6%)	0.5196	0.052
	False	26 (5.4%)	10 (4.2%)	16 (6.6%)		
	No opinion	144 (30.1%)	72 (30.5%)	72 (29.8%)		
Oils and olive oil contain a lot of cholesterol	True	168 (35.1%)	108 (45.8%)	60 (24.8%)	<0.0001	0.354
	False	142 (29.7%)	32 (13.6%)	110 (45.5%)		
	No opinion	168 (35.1%)	96 (40.7%)	72 (29.8%)		
Whole meal bread contains more fibre than white bread	True	374 (78.2%)	184 (78.0%)	190 (78.5%)	0.3505	0.066
	False	22 (4.6%)	14 (5.9%)	8 (3.3%)		
	No opinion	82 (17.2%)	38 (16.1%)	44 (18.2%)		
Fruit and vegetables are a source of "empty calories"	True	46 (9.6%)	32 (13.6%)	14 (5.8%)	0.0003	0.186
	False	384 (80.3%)	172 (72.9%)	212 (87.6%)		
	No opinion	48 (10.0%)	32 (13.6%)	16 (6.6%)		
Butter and fortified margarines are high in vitamins A and D	True	172 (36.0%)	66 (28.0%)	106 (43.8%)	0.0014	0.166
	False	82 (17.2%)	44 (18.6%)	38 (15.7%)		
	No opinion	224 (46.9%)	126 (53.4%)	98 (40.5%)		
Yellow (hard) cheese is a better calcium source than cottage cheese	True	68 (14.2%)	22 (9.3%)	46 (19.0%)	0.0009	0.171
	False	224 (46.9%)	128 (54.2%)	96 (39.7%)		
	No opinion	186 (38.9%)	86 (36.4%)	100 (41.3%)		
Offal contains significant amounts of "bad" LDL cholesterol	True	158 (33.1%)	90 (38.1%)	68 (28.1%)	0.0002	0.190
	False	82 (17.2%)	24 (10.2%)	58 (24.0%)		
	No opinion	238 (49.8%)	122 (51.7%)	116 (47.9%)		
Complex carbohydrates should be replaced with simple sugars	True	80 (16.7%)	50 (21.2%)	30 (12.4%)	<0.0001	0.230
	False	266 (55.6%)	104 (44.1%)	162 (66.9%)		
	No opinion	132 (27.6%)	82 (34.7%)	50 (20.7%)		
Protein should be the primary energy source in a proper diet	True	242 (50.6%)	126 (53.4%)	116 (47.9%)	0.0263	0.123
	False	110 (23.0%)	42 (17.8%)	68 (28.1%)		
	No opinion	126 (26.4%)	68 (28.8%)	58 (24.0%)		
Insufficient vitamin PP intake may cause dermatitis and diarrhoea	True	118 (24.7%)	52 (22.0%)	66 (27.3%)	0.0020	0.161
	False	28 (5.9%)	6 (2.5%)	22 (9.1%)		
	No opinion	332 (69.5%)	178 (75.4%)	154 (63.6%)		
	True	406 (84.9%)	192 (81.4%)	214 (88.4%)	0.0928	0.100

Sun exposure promotes vitamin D production	False	22 (4.6%)	14 (5.9%)	8 (3.3%)		
	No opinion	50 (10.5%)	30 (12.7%)	20 (8.3%)		
Phosphorus is a component of nervous tissue	True	116 (24.3%)	46 (19.5%)	70 (28.9%)		
	False	46 (9.6%)	22 (9.3%)	24 (9.9%)	0.0441	0.114
	No opinion	316 (66.1%)	168 (71.2%)	148 (61.2%)		
The calcium-to-phosphorus ratio in a proper diet should be 1:1	True	70 (14.6%)	34 (14.4%)	36 (14.9%)		
	False	52 (10.9%)	24 (10.2%)	28 (11.6%)	0.8652	0.025
	No opinion	356 (74.5%)	178 (75.4%)	178 (73.6%)		
Eating vitamin C-rich fruit increases iron absorption	True	246 (51.5%)	102 (43.2%)	144 (59.5%)		
	False	28 (5.9%)	14 (5.9%)	14 (5.8%)	0.0012	0.168
	No opinion	204 (42.7%)	120 (50.8%)	84 (34.7%)		
Starting to cook vegetables in cold water helps preserve nutrients	True	158 (33.1%)	82 (34.7%)	76 (31.4%)		
	False	86 (18.0%)	24 (10.2%)	62 (25.6%)	<0.0001	0.204
	No opinion	234 (49.0%)	130 (55.1%)	104 (43.0%)		

Adolescents: n = 236; Young adults: n = 242. Values are n (%). p-values are from chi-square tests; effect size is Cramer's V.

Table S8. Health beliefs and self-rated mental well-being by age group.

Item	Response	Total n (%)	Adolescents n (%)	Young adults n (%)
Self-rated mental well-being	Good	278 (58.2%)	130 (55.1%)	148 (61.2%)
Self-rated mental well-being	Unsatisfactory	94 (19.7%)	50 (21.2%)	44 (18.2%)
Self-rated mental well-being	Very good	66 (13.8%)	36 (15.3%)	30 (12.4%)
Self-rated mental well-being	Poor	30 (6.3%)	16 (6.8%)	14 (5.8%)
Self-rated mental well-being	Very poor	10 (2.1%)	4 (1.7%)	6 (2.5%)
Dietary habits influence disease prevention/development	Yes	428 (89.5%)	204 (86.4%)	224 (92.6%)
Dietary habits influence disease prevention/development	No	50 (10.5%)	32 (13.6%)	18 (7.4%)
Dietary habits influence disease prevention/development	Don't know	0 (0.0%)	0 (0.0%)	0 (0.0%)
Mental health is important for maintaining a healthy lifestyle	Yes	462 (96.7%)	228 (96.6%)	234 (96.7%)
Mental health is important for maintaining a healthy lifestyle	No	16 (3.3%)	8 (3.4%)	8 (3.3%)
Mental health is important for maintaining a healthy lifestyle	Don't know	0 (0.0%)	0 (0.0%)	0 (0.0%)
Moderate physical activity may alleviate depressive symptoms	Yes	362 (75.7%)	178 (75.4%)	184 (76.0%)
Moderate physical activity may alleviate depressive symptoms	No	28 (5.9%)	18 (7.6%)	10 (4.1%)
Moderate physical activity may alleviate depressive symptoms	Don't know	88 (18.4%)	40 (16.9%)	48 (19.8%)
Foods may help alleviate depressive symptoms	Yes	356 (74.5%)	180 (76.3%)	176 (72.7%)
Foods may help alleviate depressive symptoms	No	122 (25.5%)	56 (23.7%)	66 (27.3%)
Foods may help alleviate depressive symptoms	Don't know	0 (0.0%)	0 (0.0%)	0 (0.0%)
What we eat affects mood/emotions	Yes	452 (94.6%)	220 (93.2%)	232 (95.9%)

What we eat affects mood/emotions	No	26 (5.4%)	16 (6.8%)	10 (4.1%)
What we eat affects mood/emotions	Don't know	0 (0.0%)	0 (0.0%)	0 (0.0%)

Notes: Values are presented as n (%). Adolescents: ≤19 years; young adults: 20–30 years.

Table S9. Depression literacy and symptom recognition in adolescents and young adults.

A. Depression literacy indicators (by age group)

Item	Response	Total n (%)	Adolescents n (%)	Young adults n (%)	p (χ^2); V
Able to recognise depression in self/others	Yes	242 (50.6%)	130 (55.1%)	112 (46.3%)	0.0711; V=0.105
Able to recognise depression in self/others	No	44 (9.2%)	16 (6.8%)	28 (11.6%)	
Able to recognise depression in self/others	Don't know	192 (40.2%)	90 (38.1%)	102 (42.1%)	
Depression perceived as a public health problem (Poland)	Yes	366 (76.6%)	186 (78.8%)	180 (74.4%)	0.5178; V=0.052
Depression perceived as a public health problem (Poland)	No	22 (4.6%)	10 (4.2%)	12 (5.0%)	
Depression perceived as a public health problem (Poland)	Don't know	90 (18.8%)	40 (16.9%)	50 (20.7%)	

B. Symptom categories reported in open-ended responses (coded; N = 478)

Symptom category	n (%)
apathy/anhedonia	374 (78.2%)
sadness/low mood	356 (74.5%)
sleep problems	168 (35.1%)
fatigue	126 (26.4%)
suicidal thoughts	88 (18.4%)
anxiety	86 (18.0%)
concentration	44 (9.2%)
appetite/weight	42 (8.8%)
somatic/GI	14 (2.9%)

Notes: Values are presented as n (%). Adolescents: ≤19 years; young adults: 20–30 years. Block A p-values are from χ^2 tests comparing adolescents vs young adults. Block B is based on coded open-ended responses; categories are not mutually exclusive (multiple themes could be present in a single response).

Table S10. Sex-stratified sensitivity analyses for the supplementation model (yes/sometimes vs. no)

Predictor	Women OR (95% CI)	p	Men OR (95% CI)	*p*
Age (per 1 year)	1.05 (1.00–1.10)	0.0484	0.97 (0.92–1.03)	0.3853
Tertiary education (ref. lower)	1.13 (0.42–3.03)	0.8025	0.45 (0.14–1.50)	0.1958
Nutrition knowledge (per 1 point)	1.05 (0.95–1.16)	0.3385	1.11 (1.01–1.22)	0.0388
DQI (per 1 point)	1.01 (0.99–1.03)	0.3902	0.99 (0.96–1.01)	0.2458
Social media as an information source (yes vs. no)	2.21 (1.23–3.99)	0.0083	2.57 (1.18–5.61)	0.0174

Notes: *Sex-stratified logistic regression models were performed as sensitivity analyses for the primary supplementation outcome (yes/sometimes vs. no). OR = odds ratio; CI = confidence interval; DQI = diet quality index.*

Table S11. Self-reported chronic conditions in the study sample (N = 478).

Condition category	n	% of total sample
Any chronic condition	77	16.1
Thyroid disorders	30	6.3
Respiratory/allergic disorders	15	3.1
Hypertension	10	2.1
Diabetes or insulin resistance	8	1.7
Gastrointestinal disorders	6	1.3
Mental health disorders	4	0.8

Notes: *Categories were derived from coding of self-reported open-ended responses. Categories were not mutually exclusive; a single participant could report more than one chronic condition.*
